# BCKDK regulates breast cancer cell adhesion and tumor metastasis by inhibiting TRIM21 ubiquitinate talin1

**DOI:** 10.1038/s41419-023-05944-4

**Published:** 2023-07-17

**Authors:** Chunlan Xu, Kunao Yang, Zuodong Xuan, Jinxin Li, Yankuo Liu, Yue Zhao, Zeyuan Zheng, Yang Bai, Zhiyuan Shi, Chen Shao, Lei Zhang, Huimin Sun

**Affiliations:** 1 School of Medicine, Xiamen University, 361102 Xiamen, China; 2 Department of Urology, Xiang’an Hospital of Xiamen University, School of Medicine, Xiamen University, 361102 Xiamen, China; 3 School of Public Health, Xiamen University, Xiamen, China; 4 Central Laboratory, Xiang’an Hospital of Xiamen University, School of Medicine, Xiamen University, 361101 Xiamen, China

**Keywords:** Tumour biomarkers, Metastasis

## Abstract

Breast cancer is the most common malignant cancer in women worldwide. Cancer metastasis is the major cause of cancer-related deaths. BCKDK is associated with various diseases, including proliferation, migration, and invasion in multiple types of human cancers. However, the relevance of BCKDK to the development and progression of breast cancers and its function is unclear. This study found that BCKDK was overexpressed in breast cancer, associated with poor prognosis, and implicated in tumor metastasis. The downregulation of BCKDK expression inhibited the migration of human breast cancer cells in vitro and diminished lung metastasis in vivo. BCKDK perturbed the cadherin-catenin complex at the adherens junctions (AJs) and assembled focal adhesions (FAs) onto the extracellular matrix, thereby promoting the directed migration of breast cancer cells. We observed that BCKDK acted as a conserved regulator of the ubiquitination of cytoskeletal protein talin1 and the activation of the FAK/MAPK pathway. Further studies revealed that BCKDK inhibited the binding of talin1 to E3 ubiquitin ligase-TRIM21, leading to the decreased ubiquitination/degradation of talin1. In conclusion, identifying BCKDK as a biomarker for breast cancer metastasis facilitated further research on diagnostic biomarkers. Elucidating the mechanism by which BCKDK exerted its biological effect could provide a new theoretical basis for developing new markers for breast cancer metastasis and contribute to developing new therapies for the clinical treatment of breast cancer patients.

## Introduction

Breast cancer is the most common female malignancy. Localized breast cancer has a 99% 5-year relative survival rate, which dramatically drops to 28% in patients with distant metastasis [1]. Metastasis, a process of cancer cells spreading from the primary tumor to distant organs, is the defining feature of malignant tumor cells and the major contributor to mortality in cancer patients [2, 3]. Breast cancer remains the leading cause of cancer deaths globally despite the advances in screening, diagnosis, and treatment [4].

Metastasis is a multistep sequential process in which cell adhesion is essential [5]. Cell adhesion refers to those between cells and those between cells and their extracellular matrix (ECM). Cell adhesion is essential for numerous physiological processes, such as embryonic development, cell migration, tissue remodeling, and other dynamic equilibrium, which is the molecular basis of neoplastic cells prone to invasion and metastasis, the biological phenomenon through which a cell maintains its morphology and function and, a way for cells to communicate [6, 7].

Branched-chain keto-acid dehydrogenase kinase (BCKDK) is the rate-limiting enzyme in the metabolic pathway of branched-chain amino acids (BCAAs), which phosphorylates the E1α subunit of BCKDH (BCKDHA) to inhibit activity [8]. A dysfunction of BCKDK is closely associated with various diseases, such as Huntington’s disease (HD) [9], autism [10], and obesity [11].

Recently, the overexpression of BCKDK has been linked to tumor progression in various cancers. BCKDK has been depicted to induce constitutive MEK-ERK signaling cascade activation, promoting multiple tumor cell proliferation, invasion, and migration, including colorectal [12], liver [13], and ovarian cancer [14]. A study reported that adhesion-associated signaling pathways are downregulated after BCKDK knockdown and that BCKDK is closely related to talin1. BCKDK was reported to be involved in mtorc1 signal transduction, protein translation, and mitochondrial function in triple-negative breast cancer (TNBC). The downregulation of BCKDK inhibits cell proliferation, prevents protein synthesis, promotes apoptosis, and enhances DOX-mediated cell death in TNBC [15]. However, as yet, the potential mechanisms of action of BCKDK in cancer progression and metastasis are unclear.

Talin1 is a cytoskeletal protein and a key component of FA complexes. It is essential for integrin activation and the formation of focal adhesions. A loss of talin1 impairs EMT and decreases cell motility [16], and its overexpression is associated with the malignant biology of various human malignancies, including colorectal cancer [17], prostate cancer [18], triple-negative breast cancer [19], and glioblastoma [20].

Adherens junctions (AJs) are required for the initiation and maintenance of intercellular adhesion, which plays a central role in regulating the pathway of the behaviors of epithelial cells, including cell proliferation, migration, polarization, and survival [21]. Focal adhesions (FAs) are a subcellular architecture that is mainly located in the cell membrane and the extracellular matrix and as a molecular scaffold coordinating different signaling pathways and exerting traction forces on the ECM [22]. Many of the essential components of FA are tyrosine kinase and its substrates, where focal adhesion kinase (FAK) has been demonstrated to participate mainly in FA dynamics and regulate the process of the assembly and disassembly of FA complexes [23]. FAK regulates cell migration and invasion via its interaction with SRC, integrin, and the growth factor receptor signaling pathway and exerts oncogenic effects in multiple human cancers. Previous studies have reported a significant correlation between the activation of the FAK pathway and breast cancer metastasis [24, 25].

This study determined the potential relationship between BCKDK overexpression in clinical breast tumor specimens and metastasis. Through in vitro experiments, we found that BCKDK profoundly promoted the activation of FAK in the breast cancer cell lines and the changes in the cell adhesion and further validated the BCKDK effects on forming FA complexes via the regulating talin1. Using timsTOF Pro mass spectrometry, we identified that TRIM21 was an E3 ubiquitin ligase that mediated the talin1 ubiquitination. By affecting the talin1 binding to TRIM21, BCKDK inhibited the talin1 ubiquitination and degradation, promoting the activation of the FAK/MAPK signaling pathways and the assembly of new FA structures, thereby enhancing the tumor cell migration. Hence, this study revealed the mechanism of BCKDK regulating cell adhesion and breast cancer metastasis.

## Results

### High BCKDK expression correlated with poor prognosis of breast cancer patients

**Fig. 1:**
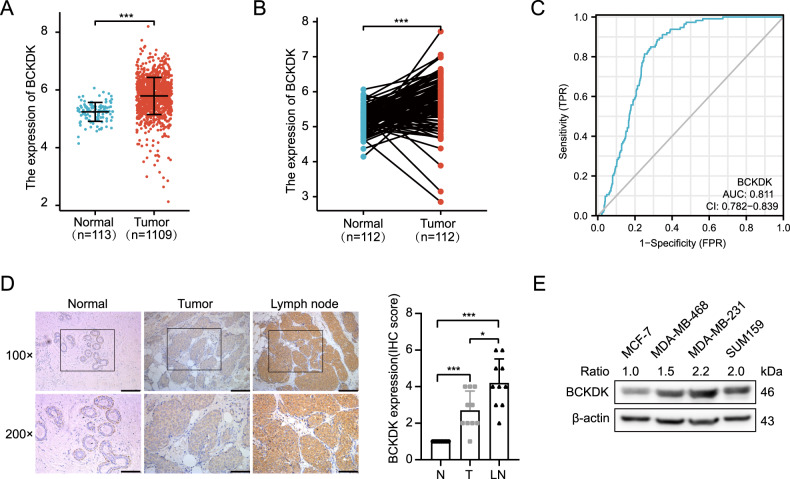
BCKDK is upregulated in BRCA and associated with poor prognosis in BRCA patients. **A** The mRNA expression of BCKDK in normal and tumor tissues in the TCGA database; the analysis was performed by the R project. Error bars represent the mean ± SD values. **B** The paired differential gene expression between normal and BRCA tumor tissue. **C** The predictive power of BCKDK to predict tumor and normal outcomes. The closer to 1 the AUC value, the better the predictive power (AUC = 0.811, CI = 0.782–0.839). **D** Immunohistochemical analysis of BCKDK expression in breast cancer tumors. Representative BCKDK immunohistochemical staining images in serial sections from the same tumor tissue samples and axillary lymph node metastases tissue, scale bar 100 μm. **E** BCKDK expression in the indicated four breast cancer cell lines was analyzed by western blot.

We analyzed the mRNA expression of BCAAs catabolism enzymes in the TCGA breast cancer RNA-seq dataset. We found significant differences in BCAT, PP2C, BCKDK, and KLF15 at the mRNA level and consistent with a previous study (Fig. 1A, B, Supplementary Fig. S1A). Since the impact of BCKDK on TNBC metabolism and proliferation was examined in prior studies, we focused on studying the role of BCKDK in the adhesion and metastasis of breast cancer cells. BCKDK exhibited a certain accuracy of predictions for predicting tumor and normal outcomes (Fig. 1C). To determine the relationship between the BCKDK expression and the metastasis in breast cancer tissues, we obtained 10 tumor specimens, matched adjacent tissues, and axillary lymph node metastases, and assessed the BCKDK protein using IHC. Lymph node metastases had significantly higher BCKDK levels than their BRCA primaries, while the adjacent normal tissue was the tissue with the lowest expression (Fig. 1D). To investigate BCKDK’s specific role in different malignant breast cancer cells, we examined the BCKDK protein expression of different breast cancer cell lines by western blot. We found that the highly aggressive and metastatic triple-negative breast cancer cell lines MDA-MB-231, MDA-MB-468, and SUM159 possessed higher levels of the BCKDK protein, but these levels were considerably lower in the MCF-7 cell lines (Fig. 1E). Previous studies have reported MCF-7 and MDA-MB-231 cell lines with low and high metastatic potentials, respectively [26]. Accordingly, we chose the MDA-MB-231 and MCF-7 cell lines for further study (Supplementary Fig. S1B). The abovementioned results indicated that BCKDK was a valuable prognostic marker that might play an important role in the development of breast cancer.

### BCKDK could induce cell migration, FAs assembly, and AJs dissociation in breast cancer cell lines

**Fig. 2:**
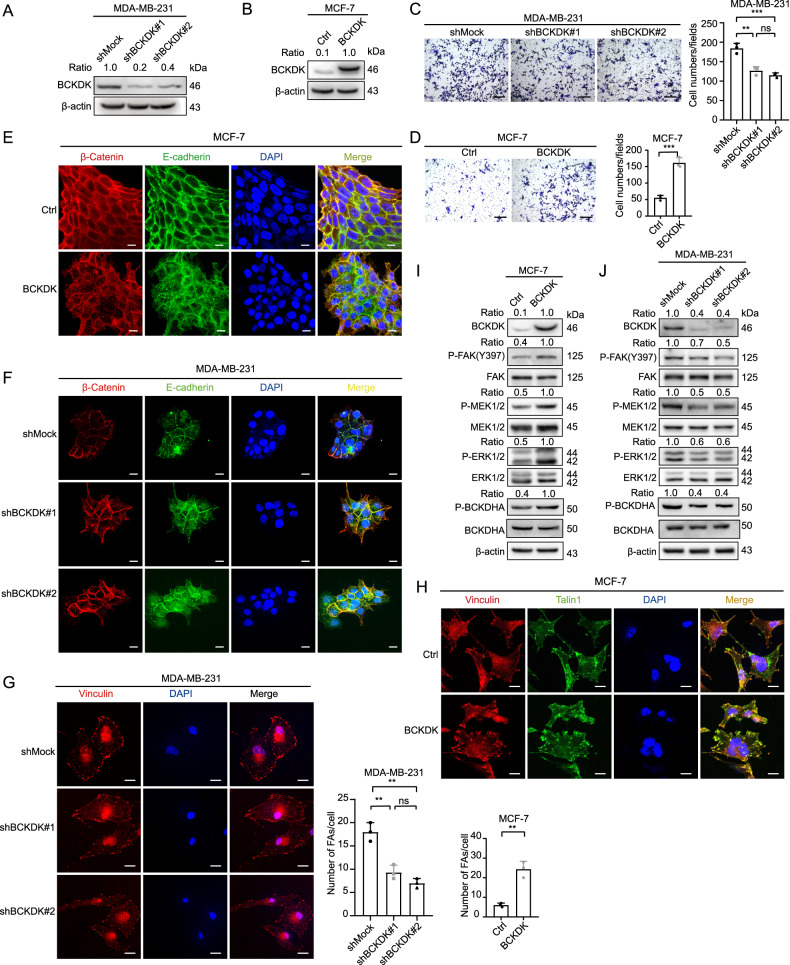
BCKDK was able to promote dissociation of AJs, FA formation, and cell migration in breast cancer cell lines through inducing FAK/MAPK signal pathway. **A** BCKDK was overexpressed in MCF-7 cell lines. **B** BCKDK was knocked down in MDA-MB-231 cell lines. **C**, **D** The migration capability of MCF-7 and MDA-MB-231 cells was detected by transwell assay; data were represented as means ± SD of triplicate experiments. ^*^, means *P* < 0. 05, ^**^, means *P* < 0. 01, ^***^, means *P* < 0. 001. Scale bar: 100 μm. **E**, **F** The integrity of AJs was visualized by IF assay in both cells. AJs were visualized by E-cadherin (green) and β-catenin (red). Nuclei were counterstained with DAPI (blue). Scale bar: 20 μm. **G**, **H** The number of focal adhesions in both cells was visualized by the IF assay. FAs were visualized by Vinculin (red) and talin1 (green). Nuclei were counterstained with DAPI (blue). Scale bar: 20 μm. **I**, **J** The expression of FAK/MAPK signal pathway markers was detected by western blot.

To explore whether BCKDK could promote the malignant biological behaviors of breast cancer cells, we obtained MDA-MB-231 cells with a stable reduction in the BCKDK expression by using short hairpin RNA (shRNA) lentiviral constructs (shBCKDK(#1/#2)) (Fig. 2A). Concurrently, we generated MCF-7 cells, which stably overexpressed BCKDK (Fig. 2B). Wound healing assays and transwell assays indicated that the knockdown of BCKDK significantly inhibited cell migration (Fig. 2C, Supplementary Fig. S2A). In contrast, it promoted the migration of BCKDK, overexpressing the MCF-7 cells (Fig. 2D, Supplementary Fig. S2B). Previous studies have reported that BCKDK promotes epithelial-to-mesenchymal transition (EMT) in colorectal cancer [27], which is related to the promotion of cancer cell adhesion and its motility. We found that BCKDK can also upregulate the expression of EMT-related molecular markers in breast cancer (Supplementary Fig. S2C, D). The disintegration of the intercellular junctions is also a sign of EMT. An overexpression of BCKDK disrupted the cell AJs of MCF-7 cells while following the knockdown of the BCKDK-enhanced cell junction formation (Fig. 2E, F). Concurrently, we found that the number of FAs was positively correlated with the level of BCKDK expression (Fig. 2G, H). Some studies have reported that the FAK and MAPK signaling pathway activation contributes to the promotion of cell migration and motility [28]. BCKDHA is a known substrate of BCKDK, and p-BCKDHA (S293) was detected by western blot to reflect the activity of BCKDK. Consistent with previous reports, we treated C57BL/6 female mice with BT2 (a small-molecule inhibitor of BCKDK) for 1 week, which increased liver BCKDH enzyme activity and decreased plasma BCAA levels (Supplementary Fig. S2E, F). In addition, the concentration of BCAA decreased in cell supernatant collection after BCKDK knockdown and increased after overexpressing BCKDK levels (Supplementary Fig. S2G, H). The BCKDK overexpression led to increased FAK (Y397), MAPK kinase (MEK1/2), and ERK1/2 phosphorylation (Fig. 2I, J). These results indicated that BCKDK weakened the AJs and increased the number of FAs and their migration capacity for breast cancer cells.

### Expression of BCKDK affects the talin1 expression levels in breast cancer cells

**Fig. 3:**
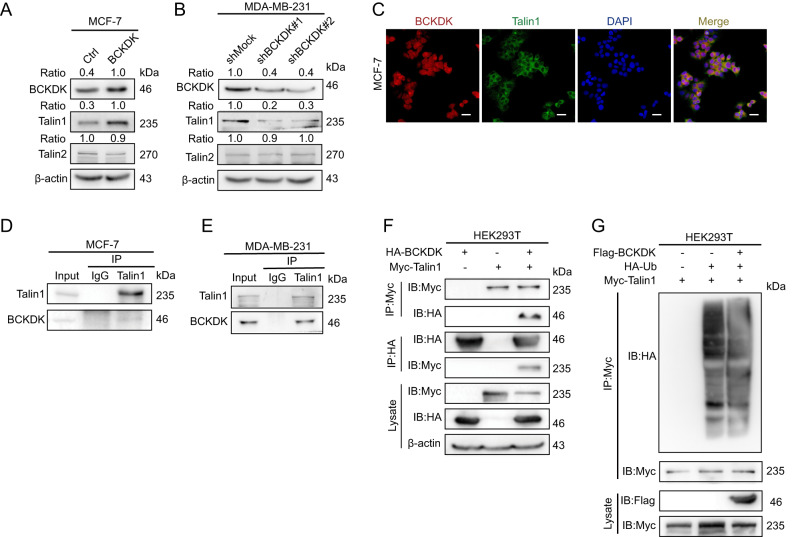
Expression of BCKDK affects the talin1 expression levels in breast cancer cells. **A**, **B** The expression of talin1 and talin2 were detected by western blot in cells of BCKDK overexpression or knockdown. **C** IF assay showed the colocalization of talin1 and BCKDK in MCF-7 cells. Cells were stained for BCKDK (red) and talin1 (green). Scale bar, 50 μm. **D**, **E** BCKDK was coimmunoprecipitated with talin1 in MCF-7 and MDA-MB-231 cells. **F** Exogenous Talin1 coimmunoprecipitated with BCKDK in HEK293T cells. **G** The ubiquitination of talin1 was examined by western blot in HEK293T cells.

A BCKDK overexpression correlated with the formation of FA and increased EMT activation. To further investigate the mechanism by which BCKDK promotes the dissociation of AJs, the formation of FAs, and the migration of breast cancer cells, we carefully measured the protein levels of talin1 and talin2 when overexpressing BCKDK by western blotting and found that the BCKDK expression levels were significantly upregulated and that the knockdown of BCKDK decreased talin1 (Fig. 3A, B). This indicated a positive regulatory relationship between BCKDK and talin1. There is no significant difference in talin2. Immunofluorescence experiments demonstrated that BCKDK and talin1 were co-localized in the cell cytoplasm and the cell membrane (Fig. 3C). When the MCF-7 and MDA-MB-231 cells were lysed and processed for immunoprecipitation (IP) with anti-talin1 antibodies respectively, we saw the co-immunoprecipitation (CO-IP) of BCKDK, indicating that talin1 and BCKDK interacted endogenously (Fig. 3D, E). In addition, talin1-Myc and BCKDK-HA were transfected individually or together into the HEK293T cells. Following transfection, the anti-HA or anti-Myc antibodies were used to perform CO-IP from the lysates of the transfected HEK293T cells. The results suggested that the talin1 co-precipitated with BCKDK (Fig. 3F). These results indicated that BCKDK interacted with talin1 in breast cancer cells. Next, we investigated whether BCKDK affected the stability of the talin1 proteins. We found that the BCKDK overexpression increased the talin1 ubiquitination (Fig. 3G).

### BCKDK competitively interacted with talin1 to disrupt the TRIM21 binding to talin1 and inhibited the talin1 ubiquitination degradation

**Fig. 4:**
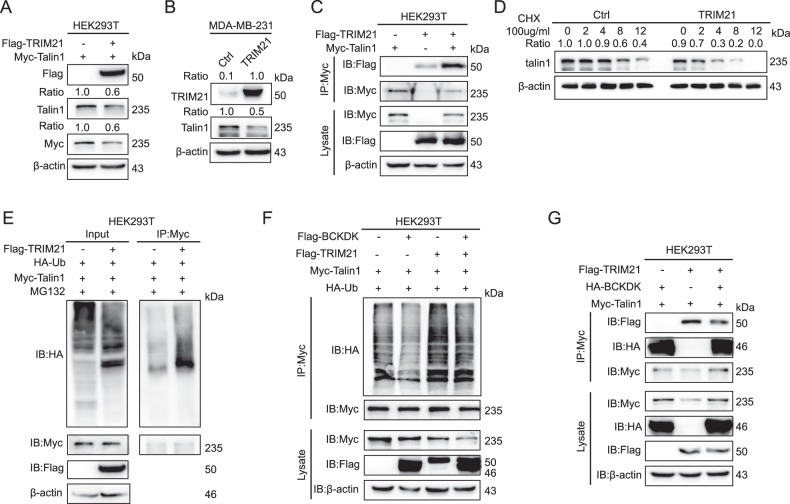
BCKDK competitively interacts with talin1 to disrupt TRIM21 binding to talin1 and inhibit talin1 ubiquitination degradation. **A** The expression of talin1 was examined by western blot in HEK293T cells. **B** The expression of talin1 was examined by western blot in MDA-MB-231 cells. **C** Exogenous talin1 coimmunoprecipitated with TRIM21 in HEK293T cells. **D** The degradation protein level of talin1 detected in M231/Ctrl and M231/TRIM21 cells with CHX treatment (100 μg/mL) for indicated time intervals. **E** Co-IP shows that HA-ubiquitin is pulled down by talin1 in HEK293T cells. HEK293T cells were transfected with Myc-tagged talin1 and HA-ubiquitin, plus Flag-tagged TRIM21 or the vector controls. At 48 h post-transfection, cells were treated with MG132 for 4 h, and then lysates of these cells were subjected to IP with anti-Myc-conjugated beads. Immunoprecipitates were subjected to IB analysis for ubiquitinated talin1 with anti-ubiquitin or for indicated proteins. **F** HEK293T cells were transfected with Myc-tagged talin1 and HA-ubiquitin, plus Flag-tagged TRIM21, Flag-tagged BCKDK or their vector controls. At 48 h post-transfection, cells were lysed and subjected to IP with anti-Myc-conjugated beads. The immunoprecipitates were subjected to IB analysis for ubiquitinated talin1 with anti-ubiquitin or for indicated proteins. **G** IP analysis using anti-Myc antibody followed by IB analysis to detect HA-BCKDK and Flag-TRIM21 expression in HEK293T cells.

The ubiquitination-mediated degradation of talin1 required specific E3 ubiquitin ligases. We speculated that BCKDK affected the expression of talin1 by affecting its E3 ubiquitin ligase. To identify the potential E3 ubiquitin ligase of talin1, we screened out TRIM21 as the highest-score E3 ligase of talin1 immunoprecipitations by using the timsTOF Pro mass spectrometry techniques (Supplementary Fig. S3A). To evaluate whether TRIM21 led to the ubiquitination of talin1, we overexpressed exogenous TRIM21-Flag in the HEK293T cells and treated the cells with MG132 to prevent protein degradation before performing a ubiquitination assay. The results demonstrated the downregulation of the talin1 protein level (Fig. 4A). Similarly, the overexpression of TRIM21 in the MDA-MB-231 cells led to a decrease in talin1 (Fig. 4B). As the TRIM21 overexpression-mediated downregulation of the talin1 protein level seemed to occur through the proteasomal degradation pathway, we examined whether the two proteins combined. Immunoprecipitation experiments of the exogenous talin1-Myc and TRIM21-Flag proteins co-expressed in HEK293T cells were performed, and the anti-Myc pulled down the Flag, indicating that talin1 and the TRIM21 protein interacted with each other (Fig. 4C). We treated the cells with the protein synthesis inhibitor CHX, and then, measured the talin1 levels at various time points by western blotting. the talin1 protein half-life was shortened by the TRIM21 overexpression (Fig. 4D). Next, we validated that TRIM21 enhanced the ubiquitination of talin1 (Fig. 4E). These data suggested that TRIM21 might induce ubiquitination with the subsequent proteasomal degradation of talin1. After that, we wondered whether BCKDK affected the expression levels of TRIM21 and thereby influenced the talin1 expression. The results revealed that the BCKDK overexpression did not affect the TRIM21 expression (Supplementary Fig. S3B), but the TRIM21-mediated ubiquitination of talin1 was significantly inhibited (Fig. 4F). Therefore, we considered that BCKDK might affect the TRIM21-mediated ubiquitination of talin1. As both BCKDK and TRIM21 could bind to talin1. In the immunoprecipitation experiments, we found that the BCKDK overexpression could weaken the interaction of TRIM21 with talin1 (Fig. 4G). However, in order to exclude the influence of trim21, we detected the expression of AKT, p-AKT, and JAK2 after altering BCKDK and found no significant differences in this pathway (Supplementary Fig. S3C, D). These results indicated that BCKDK upregulated talin1 by blocking the TRIM21-mediated ubiquitination.

### BCKDK activates FAK/MAPK signaling pathway through talin1

**Fig. 5:**
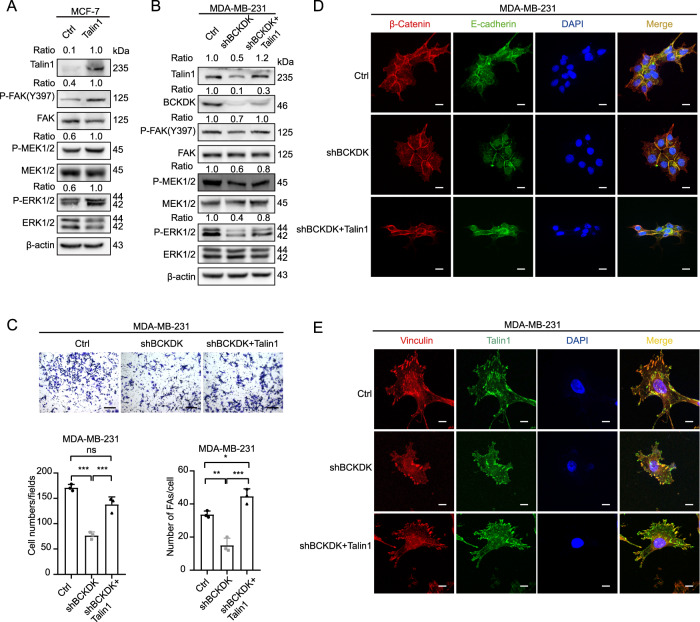
BCKDK activates FAK/MAPK pathway through talin1. **A**, **B** The expression of FAK/MAPK signal pathway markers was detected by western blot in these cell lines. **C** The migration capability of MDA-MB-231 cells was detected by transwell assay; data were represented as means ± SD of triplicate experiments. ^*^, means *P* < 0. 05, ^**^, means *P* < 0. 01, ^***^, means *P* < 0. 001. Scale bar: 100 μm. **D** The integrity of AJs was visualized by IF assay in MDA-MB-231 cells. AJs were visualized by E-cadherin (green) and β-catenin (red). Nuclei were counterstained with DAPI (blue). Scale bar: 20 μm. **E** The number of focal adhesions in MDA-MB-231 cells was visualized by IF assay. FAs were visualized by Vinculin (red) and talin1 (green). Nuclei were counterstained with DAPI (blue). Scale bar: 20 μm.

To further validate the role of the BCKDK/talin1 axis in the FAK/MAPK signaling, we overexpressed talin1 in the MCF-7 cells. As expected, talin1 could induce the phosphorylation of FAK (Y397), MEK1/2, and ERK1/2 (Fig. 5A). Based on the BCKDK knockdown, the overexpression of talin1 restored the phosphorylation level of FAK (Y397), MEK1/2, and ERK1/2 partially in the MDA-MB-231 cells (Fig. 5B). The transwell assay and the immunofluorescence experiment depicted that the talin1 overexpression could abolish the effect of the BCKDK knockdown on the cell migration capacity, AJ integrity, and assembly of FAs (Fig. 5C–E). In summary, the BCKDK/talin1 axis led to the dissociation of AJs, and the formation of FAs increased because of the activation of the FAK/MAPK signaling pathway, which further promoted breast cancer cell migration. In addition, we found that BCKDK promoted the talin1 combination with β1-integrin, while the BCKDK knockdown attenuated the binding (Supplementary Fig. S4A, B).

### BCKDK promotes breast cancer metastasis in vivo

**Fig. 6:**
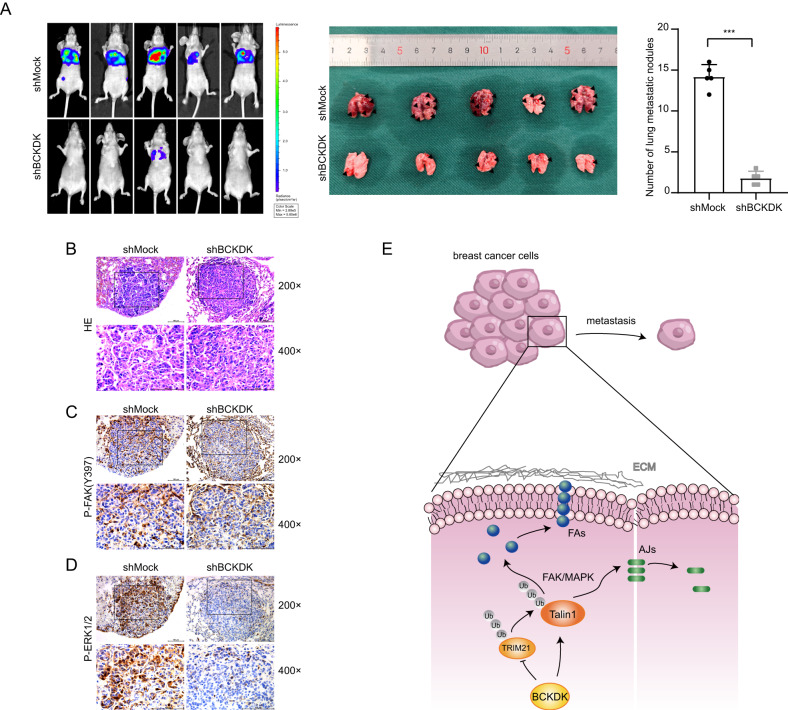
BCKDK promotes BRCA metastasis in vivo. **A** Luciferase-labeled MDA-MB-231 (MDA-MB-231-Luciferase) cells were injected into 10 female nude mice via tail vein injection. Lung metastasis was monitored by bioluminescence using an in vivo imaging system in the left panel. Number of metastatic nodules in the lung. Error bars represent the mean ± SD. (^*^means *P* < 0. 001). **B** H&E staining of lungs from the indicated groups upon harvest (day 21). Scale bars, 100 µm. **C**, **D** The expression of p-FAK and p-ERK1/2 in nude mice lung tissue was detected by immunohistochemistry. Scale bars, 100 µm. **E** The schematic diagram of the role of BCKDK/talin1 signaling axis in affecting the adhesion mechanism of breast cancer cells.

Finally, to validate the metastatic potential of BCKDK in vivo, we performed the tail vein injection of the control and BCKDK knockdown Luciferase-labeled MDA-MB-231 (MDA-MB-231-Luciferase) cells in nude mice (10 animals). These in vivo results were consistent with those of the in vitro experiments. BCKDK knockdown decreased the metastatic potential of the tumor cells to a certain degree. We found that the BCKDK knockdown significantly decreased the metastases of tumor cells to the lung after the tail vein injection compared to the control; metastatic nodules were counted by visual observation (Fig. 6A). The lungs were harvested and stained with hematoxylin and eosin (H&E) to identify the metastatic nodules (Fig. 6B). The lung tissues were analyzed by immunohistochemistry, and the results revealed that the BCKDK knockdown reduced the p-FAK (Y397) and p-ERK1/2 protein levels (Fig. 6C, D). Together, these mouse models strongly indicated a metastasis-promoting role for BCKDK in breast cancer.

Taken together, our findings indicated that BCKDK inhibited the talin1 ubiquitination and degradation by blocking the binding of the E3 ubiquitin ligase to talin1. The BCKDK/talin1 axis, through the activation of the FAK/MAPK signaling pathways, perturbed the AJs and regulated the formation and maturation of FAs, thereby inducing EMT and promoting breast cancer metastasis (Fig. 6E).

## Discussion

During the process of cell migration, its morphology and molecular changes occur to obtain mesenchymal phenotypes, including loss of intercellular connections, recombination of actin cytoskeleton, reprogramming of gene expression, and separation from surrounding cells and ECM [29]. The structures that form and maintain the cell–cell contacts include adherens junctions (AJs) and tight junctions (TJs). AJs are required for the initiation and maintenance of intercellular adhesion, which plays a central role in regulating the pathway of the behaviors of epithelial cells, including cell proliferation, migration, polarization, and survival. Within the AJ complex, the E-cadherin extracellular repeat domain forms calcium-dependent trans interactions between two adjacent cells, and the cytosolic tail of E-cadherin is linked to the actin cytoskeleton through bound catenin proteins (α-, β-, and p120-catenin). AJ is the major cell–cell adhesion form with remarkable plasticity in epithelial cells, and epithelial E-cadherin continuously turns over and undergoes a cycle of endocytosis, sorting, and recycling back to the plasma membrane, which is a prerequisite for tissue structure and morphogenesis [30]. Epithelial polarity mechanisms, particularly Par3 apical scaffolds, control the reversible assembly and disassembly of E-cadherin-based adhesion junction complexes to regulate the AJs’ localization, size, and strength [31]. E-cadherin and p120-catenin are two major components of AJs, and their downregulation is a frequent hallmark of cancer, leading to a loss of cell polarity and increased epithelial proliferation, invasion, and metastasis. In addition, the phosphorylation of the components of the AJ complex can affect the stability of the AJ. Previous studies have depicted that activated ERK1/2 phosphorylates p120-catenin through TGF-β action, thereby promoting p120-catenin binding to E3 ubiquitin ligase SMurf1, leading to AJ complex dissociation, TJ dissociation, and cytoskeletal rearrangement of tumor cells, EMT, and promotion of breast cancer metastasis [32]. Cancer cells are required to perform these rearrangements of actin, cytoskeleton, and E-cadherin-based AJs to obtain favorable epithelial/mesenchymal phenotypes and partial migration phenotypes generated by EMT for plasticity [33]. FAs involving integrins or mechanical forces exerted at the cells establish a link between ECM and the actin cytoskeleton and exert traction forces on the ECM, leading to the generation of the net pushing force to push the cells forward [22]. The dysregulation of adhesion complexes contributes to various diseases, such as cancer, diabetes, and cardiovascular disease. Extracellular matrix-associated adhesins and intercellular adhesins play crucial roles in regulating the different stages of cancer cell metastasis and determining cancer cell invasiveness [34, 35]. Tumor aggressiveness depends on the balance between E-cadherin-mediated AJs and integrin-mediated cell-ECM adhesion [36]. Studies have revealed that a dynamic cycle of FA assembly, cytoskeleton remodeling, and FA dissociation, known as focal adhesion turnover, enables cells to achieve motility [37], and FA dysregulation is a critical step in tumor invasion and metastasis.

BCAT1 and BCKDK are key enzymes in the BCAA catabolic pathway. BCAT1 is overexpressed in multiple cancer types, causing malignant phenotypes and a poor prognosis. Overexpression of BCAT1 in lung cancer cells led to a dramatic downregulation of α-KG and resulted in activation of SOX2 [38]. BCAT1 could promote gastric cancer cell invasion, proliferation, and angiogenesis through PI3K/AKT/mTOR signaling pathway [39]. In breast cancer, BCAT1 activated mTORC1 signaling, increased mitochondrial biogenesis, and thereby promoted cell growth [40]. The overexpression of BCKDK causes an excessive accumulation of BCAA, which has previously been reported to be associated with early events in pancreatic cancer development [41]. BCKDK as a relevant metabolic regulator through its actions on BCAA and citric acid, affects glycolysis and oxidative phosphorylation, thereby affecting the malignant progression of non-small-cell lung cancer [42]. Studies have reported that BCAT1 enhances autophagy mediated by mTOR via Leu metabolism, leading to decreased cisplatin sensitivity [43]. In addition, it has been reported that inhibition of BCKDK may synergize or potentiate cytotoxic effects of DOX-treated TNBC patients [15]. BCAT1 and BCKDK regulate the BCAA metabolism and cancer progression and have been identified as a marker for cancer prognosis. While it is not clear if BCKDK has been proposed to be involved in breast cancer cell metastasis via regulation of BCAT1 activity, it is of great interest to further investigate the relationship between BCKDK and BCAT1 in breast cancer development.

Previous studies have reported that BCKDK participates in the malignant progression of various tumors by activating the MAPK signaling pathway. However, the specific mechanism of BCKDK in the development of breast cancer remains unclear. In this study, we demonstrated that BCKDK was overexpressed in both clinical breast cancer specimens and breast cancer cell lines and that its expression was higher in triple-negative breast cancer with a high degree of malignancy and metastasis. Our findings demonstrated that BCKDK induced the FAK/MAPK pathway activation in breast cancer cell lines by upregulating talin1, leading to the dissociation of AJs, FA assembly, and increased cell migration. Based on the timsTOF Pro mass spectrometry results, we hypothesized that TRIM21 was an E3 ubiquitin ligase leading to the ubiquitination degradation of talin1. Further experiments proved that BCKDK inhibited the ubiquitination degradation of talin1 by inhibiting the binding of talin1 to TRIM21. The talin1 overexpression after silencing BCKDK restored the reduced FAs and the cell migration induced by the BCKDK knockdown. These findings revealed the role of a new signaling axis, BCKDK/talin1/FAK/MAPK, in regulating cell adhesion and migration, providing new insights into how BCKDK contributed to malignant tumor development. Therefore, interfering with this signaling axis might be an effective way to inhibit the invasion and metastasis of breast cancer cells.

Recent studies have demonstrated that the upregulation of the talin1 expression in triple-negative breast cancer specimens is associated with tumor metastasis and poor prognosis. Silencing talin1 can significantly inhibit tumor cell migration by interfering with the dynamic formation of the adhesion foci, thereby regulating the β-AKT signaling and EMT [19]. CDK5 has been reported to phosphorylate the talin1 head (TH) and inhibit its binding to Smurf1, thereby preventing the ubiquitination degradation of TH, leading to extensive adhesion flipping and ultimately controlling cell migration [44]. TRIM21 is a ubiquitin E3 ligase that contains the ring finger domain and plays an important role in cancer progression [45, 46]. In our study, we found that TRIM21 could interact with talin1 and promote talin1 ubiquitination degradation, suggesting that TRIM21 was the E3 ubiquitin ligase responsible for talin1 ubiquitination. In addition, we found that BCKDK significantly inhibited the TRIM21-mediated ubiquitination of talin1 and that BCKDK competitively inhibited the talin1 binding to TRIM21. It has been reported that ORAI2 promotes the dissociation of FAs at the posterior margin of gastric cancer cells by inducing FAK-mediated MEK/ERK activation and enhancing the metastatic ability of gastric cancer cells [47]. The FAK/SRC/ERK signaling pathway is essential for cell adhesion and the regulation of cell motility and migration [23, 48]. In addition, there is evidence that talin1 regulates cadherin-mediated intercellular adhesion in a β1-integrin-independent manner [49, 50]. Therefore, BCKDK might regulate the talin1 expression, activate the FAK/MAPK signaling pathway, promote FA aggregation and AJs dissociation, and then promote tumor metastasis.

The TH domain has been reported to bind to the cytoplasmic tail of the integrin beta subunit and regulate integrin activation [51]. ATG9B has been reported to be transported to the cell edge with the assistance of MYH9. ATG9B promotes colorectal cancer metastasis by mediating the interaction between β1-integrin and talin1, accelerating the FAs aggregation and promoting the β1-integrin activation [52]. In addition, talin1 phosphorylation promotes the bone metastasis of prostate cancer through the activation of the β1-integrin signaling pathway [53]. Recent studies have depicted that a small-molecule inhibitor (C67399) that blocks the interaction between talin1 and β1-integrin inhibits the tumor size and lung metastasis in vivo by interfering with the FA formation and migration in vitro may be a potential target for tumor therapy [19]. Our findings suggested that BCKDK regulated the talin1 binding to β1-integrin, but the molecular mechanism of how BCKDK regulated this process remains to be explored. BCKDK inhibitors may become future drug candidates for treating metabolic diseases and cancers caused by elevated branched-chain amino acid concentrations [54].

## Materials and methods

### Patients samples and immunohistochemistry (IHC)

Ten patient samples were obtained from Xiangan Hospital of Xiamen University. The IHC staining analysis was performed using corresponding antibodies. The immunostaining intensity was graded following the Remmele scoring method. Representative images were obtained by magnification (×100, ×200, and ×400) with an Olympus Imaging System Microscope.

### Cell culture and transfection

Human breast cancer cell lines MDA-MB-231 and MCF-7 and human embryonic kidney cell line HEK293T were purchased from American Type Culture Collection (ATCC), cultured in Dulbecco’s modified Eagles’ medium (DMEM, Gibco, Grand Island, NY) supplemented with 10% FBS and 1% penicillin/streptomycin (KeyGEN). All of the cells applied in this study were cultured at 37 °C in a humidified 5% CO_2_ atmosphere. Routine detection of mycoplasma ensures that all cells are not contaminated by mycoplasma. All the cells used in the experiment were within 15 passages, and the cells were resuscitated and passed at least three times before being used.

Plasmids were transfected using Lipofectamine 2000 (Invitrogen, USA) according to the manufacturer’s protocols. Puromycin and Hygromycin B (Spark Jade) were applied to screen the knockdown and overexpression of stable cell lines screening, respectively.

### Plasmids and shRNAs

The plasmids of pLKO.1-shBCKDK (#1-#2) were gifts from Prof. Feng Zhu. A scrambled siRNA with a sequence lacking significant homology to the human genome database was used as the control siRNA (shMOCK). pCMV3-BCKDK-HA and pCMV3-TRIM21-Flag were purchased from Sino Biological. pCMV3-BCKDK-Flag was constructed by our laboratory. pReceiver-Lv202-TLN1-Myc was customized at GeneCopoeia. An empty vector was used as the negative control.

### Transwell migration assay

Transwell plates (24-well, pore size 8 μm, Corning) were used for the transwell assay. For the migration assay, 1 × 10^5^ cells were harvested in 100 μL of a serum-free culture medium and added into the upper chamber. Next, 600 μL of 20% fetal bovine serum medium was added to the lower chamber as the chemo-attractant. After 24 h culture, the cells that crossed the inserts were fixed with 4% paraformaldehyde and stained with 0.1% crystal violet. The migrated cells were photographed and counted via an inverted microscope.

### Immunofluorescence assay

Cells grown on glass coverslips were washed three times with PBS, fixed with 4% paraformaldehyde, permeabilized with 0.25% Triton X-100 and blocked with 1% bovine serum albumin. These cells were then stained using the indicated primary and appropriately fluorescently conjugated secondary antibodies. Images were obtained using a Zeiss LSM 880 confocal microscope with the ZEN software (Carl Zeiss GmbH, Jena, Germany).

### Western blot assay and immunoprecipitation (IP)

The cells were lysed in the RIPA lysis buffer (lot: R0010, Solarbio, Inc., Beijing, CHN) containing a protease inhibitor (MedChemExpress, New Jersey, USA) and a phosphatase inhibitor (MedChemExpress, New Jersey, USA). Then, proteins were separated using 8–10% SDS-PAGE, followed by a transfer to the polyvinylidene difluoride membranes (PVDF) (Millipore, Billerica, MA, USA). Finally, the membranes were blocked with 5% non-fat milk and blotted with the corresponding primary antibodies at 4 °C overnight and detected using an enhanced chemiluminescence reagent (Thermo Fisher Scientific, MA, USA) with C300 (Azure Biosystems, CA, USA).

For CO-IP assays, an IP buffer (Thermo Fisher Scientific, MA, USA) was used to acquire the IP samples. After sonication and centrifugation, the supernatant fractions containing equal amounts of protein were immunoprecipitated by the corresponding antibody and then subjected to western blot.

### Antibodies and reagents

The BCKDK (E-12) (1:1000, sc-374425) antibodies were purchased from Santa Cruz Technology. Flag tag and talin-1 (#4021) antibodies were purchased from Sigma-Aldrich. All the following antibodies were purchased from Cell Signaling Technology: ERK1/2 (#4695), p-ERK1/2 (Thr202/Tyr204) (#4649), MEK1/2 (8727s), p-MEK1/2 (S221) (2338s), β1-integrin (D6S1W), TRIM21 (#92043), AKT (#4060), p-AKT (S473) (#13038), HA tag antibody (#3724), β-actin mouse monoclonal antibody (#3700), Anti-rabbit IgG (Alexa Fluor#488 Conjugate) (4412), Anti-mouse IgG (Alexa Fluor#594 Conjugate) (8890), secondary antibody against mouse and rabbit. The FAK (A11131), pY397-FAK (AP0302), talin-2 (A19810), and BCAT2 (A7426) antibodies were purchased from ABclonal. The vinculin (ab129002), BCKDHA (ab305168), p-BCKDHA (S293) (ab302504) antibodies were purchased from Abcam. All antibodies were used following the manufacturer’s instructions.

### Animal studies

All animals were purchased from and housed in the Laboratory Animal Center of Xiamen University (China) in a facility with 12-h light/12-h dark cycles under pathogen-free conditions. The animal studies were performed according to the guidelines approved by the Institute of Biomedical Sciences, Xiamen University. For the metastasis model, MDA-MB-231-Luciferase cells (1 × 10^6^) were intravenously injected into the tail veins of the female BALB/c athymic nude mice (6–8 weeks). The mice were sacrificed 21 days after injection, lung metastasis was monitored by the bioluminescence, and the number of lung metastasis colonies was counted. For the BCKDK inhibition study, C57BL/6 female mice (6–8 weeks) were administered the small-molecule BCKDK inhibitor (BT2, MCE) daily at a dose of 20 mg/kg dissolved in 200 µL of sterile dimethylsulfoxide by i.p. injection for 7 days (10 animals). The control group was administered an equal volume of dimethylsulfoxide each day.

### BCKDH activity assay

Tissue BCKDH activity was measured according to the manufacturer’s instructions (GENMED).

### Quantification of BCAA concentration

BCAA concentration in plasma and supernatant of cultured cells were determined using a BCAA kit (Sigma, MAK003) according to the manufacturer’s instructions.

### Statistical analysis

One-way analysis of variance (ANOVA) was used to assess the values among different experimental groups by using the GraphPad Prism program version 8. 0. 1. *P* < 0.05 was considered statistically significant. ^*^P < 0.05; ^**^P < 0.01; ^***^P < 0.001; NS, not significant. All values were presented as mean ± SD of at least triplicate experiments except for the animal experiments.

## Supplementary information

supplementary figure 1

supplementary figure 2

supplementary figure 3

supplementary figure 4

supplementary data file

Original Data File

## Data Availability

All data are available upon request.

## References

[1] Miller KD, Nogueira L, Devasia T, Mariotto AB, Yabroff KR, Jemal A, et al. Cancer treatment and survivorship statistics, 2022. CA Cancer J Clin. 2022;72:409–36. 10.3322/caac.21731 35736631

[2] Fares J, Fares MY, Khachfe HH, Salhab HA, Fares Y (2020). Molecular principles of metastasis: a hallmark of cancer revisited. Signal Transduct Target Ther.

[3] Lambert AW, Pattabiraman DR, Weinberg RA (2017). Emerging biological principles of metastasis. Cell..

[4] Weigelt B, Peterse JL, van’t Veer LJ (2005). Breast cancer metastasis: markers and models. Nat Rev Cancer.

[5] Wei SC, Yang J (2016). Forcing through tumor metastasis: the interplay between tissue rigidity and epithelial-mesenchymal transition. Trends Cell Biol.

[6] Gumbiner BM (1996). Cell adhesion: the molecular basis of tissue architecture and morphogenesis. Cell..

[7] Mui KL, Chen CS, Assoian RK (2016). The mechanical regulation of integrin-cadherin crosstalk organizes cells, signaling and forces. J Cell Sci.

[8] Suryawan A, Hawes JW, Harris RA, Shimomura Y, Jenkins AE, Hutson SM (1998). A molecular model of human branched-chain amino acid metabolism. Am J Clin Nutr.

[9] Mochel F, Charles P, Seguin F, Barritault J, Coussieu C, Perin L (2007). Early energy deficit in Huntington disease: identification of a plasma biomarker traceable during disease progression. PLoS ONE.

[10] Beaudet AL (2012). Neuroscience. Preventable forms of autism?. Science.

[11] Burrage LC, Nagamani SC, Campeau PM, Lee BH (2014). Branched-chain amino acid metabolism: from rare Mendelian diseases to more common disorders. Hum Mol Genet.

[12] Xue P, Zeng F, Duan Q, Xiao J, Liu L, Yuan P (2017). BCKDK of BCAA catabolism cross-talking with the MAPK pathway promotes tumorigenesis of colorectal cancer. EBioMedicine..

[13] Zhai M, Yang Z, Zhang C, Li J, Jia J, Zhou L (2020). APN-mediated phosphorylation of BCKDK promotes hepatocellular carcinoma metastasis and proliferation via the ERK signaling pathway. Cell Death Dis.

[14] Li H, Yu D, Li L, Xiao J, Zhu Y, Liu Y (2022). BCKDK promotes ovarian cancer proliferation and migration by activating the MEK/ERK signaling pathway. J Oncol.

[15] Biswas D, Slade L, Duffley L, Mueller N, Dao KT, Mercer A (2021). Inhibiting BCKDK in triple negative breast cancer suppresses protein translation, impairs mitochondrial function, and potentiates doxorubicin cytotoxicity. Cell Death Discov.

[16] Thapa N, Tan X, Choi S, Wise T, Anderson RA (2017). PIPKIγ and talin couple phosphoinositide and adhesion signaling to control the epithelial to mesenchymal transition. Oncogene..

[17] Ji L, Jiang F, Cui X, Qin C (2019). Talin1 knockdown prohibits the proliferation and migration of colorectal cancer cells via the EMT signaling pathway. Oncol Lett.

[18] Sakamoto S, McCann RO, Dhir R, Kyprianou N (2010). Talin1 promotes tumor invasion and metastasis via focal adhesion signaling and anoikis resistance. Cancer Res.

[19] Zhang Y, Sun L, Li H, Ai L, Ma Q, Qiao X, et al. Binding blockade between TLN1 and integrin beta1 represses triple-negative breast cancer. Elife. 2022;11:e68481. 10.7554/eLife.68481 PMC8937232 35285795

[20] de Semir D, Bezrookove V, Nosrati M, Scanlon KR, Singer E, Judkins J (2020). PHIP drives glioblastoma motility and invasion by regulating the focal adhesion complex. Proc Natl Acad Sci USA.

[21] Harris TJ, Tepass U (2010). Adherens junctions: from molecules to morphogenesis. Nat Rev Mol Cell Biol.

[22] Ridley AJ, Schwartz MA, Burridge K, Firtel RA, Ginsberg MH, Borisy G (2003). Cell migration: integrating signals from front to back. Science..

[23] Webb DJ, Donais K, Whitmore LA, Thomas SM, Turner CE, Parsons JT (2004). FAK-Src signalling through paxillin, ERK and MLCK regulates adhesion disassembly. Nat Cell Biol.

[24] Luo M, Guan JL (2010). Focal adhesion kinase: a prominent determinant in breast cancer initiation, progression and metastasis. Cancer Lett.

[25] van Nimwegen MJ, Verkoeijen S, van Buren L, Burg D, van de Water B (2005). Requirement for focal adhesion kinase in the early phase of mammary adenocarcinoma lung metastasis formation. Cancer Res.

[26] Yang H, Wang B, Wang T, Xu L, He C, Wen H (2014). Toll-like receptor 4 prompts human breast cancer cells invasiveness via lipopolysaccharide stimulation and is overexpressed in patients with lymph node metastasis. PLoS ONE.

[27] Tian Q, Yuan P, Quan C, Li M, Xiao J, Zhang L (2020). Phosphorylation of BCKDK of BCAA catabolism at Y246 by Src promotes metastasis of colorectal cancer. Oncogene..

[28] Wu WS, Wu JR, Hu CT (2008). Signal cross talks for sustained MAPK activation and cell migration: the potential role of reactive oxygen species. Cancer Metastasis Rev.

[29] Carragher NO, Frame MC (2004). Focal adhesion and actin dynamics: a place where kinases and proteases meet to promote invasion. Trends Cell Biol.

[30] Bruser L, Bogdan S. Adherens junctions on the move—membrane trafficking of E-cadherin. Cold Spring Harb Perspect Biol. 2017;9:a029140. 10.1101/cshperspect.a029140 PMC5334258 28096264

[31] Coopman P, Djiane A (2016). Adherens junction and E-cadherin complex regulation by epithelial polarity. Cell Mol Life Sci.

[32] Wu Q, Li G, Wen C, Zeng T, Fan Y, Liu C (2020). Monoubiquitination of p120-catenin is essential for TGFbeta-induced epithelial-mesenchymal transition and tumor metastasis. Sci Adv.

[33] Rubtsova SN, Zhitnyak IY, Gloushankova NA. Phenotypic plasticity of cancer cells based on remodeling of the actin cytoskeleton and adhesive structures. Int J Mol Sci. 2021;22:1821. 10.3390/ijms22041821 PMC7918886 33673054

[34] Hynes RO (2002). Integrins: bidirectional, allosteric signaling machines. Cell..

[35] Valastyan S, Weinberg RA (2011). Tumor metastasis: molecular insights and evolving paradigms. Cell..

[36] Canel M, Serrels A, Frame MC, Brunton VG (2013). E-cadherin-integrin crosstalk in cancer invasion and metastasis. J Cell Sci.

[37] Webb DJ, Parsons JT, Horwitz AF (2002). Adhesion assembly, disassembly and turnover in migrating cells—over and over and over again. Nat Cell Biol.

[38] Mao L, Chen J, Lu X, Yang C, Ding Y, Wang M (2021). Proteomic analysis of lung cancer cells reveals a critical role of BCAT1 in cancer cell metastasis. Theranostics..

[39] Shu X, Zhan PP, Sun LX, Yu L, Liu J, Sun LC (2021). BCAT1 activates PI3K/AKT/mTOR pathway and contributes to the angiogenesis and tumorigenicity of gastric cancer. Front Cell Dev Biol.

[40] Zhang L, Han J (2017). Branched-chain amino acid transaminase 1 (BCAT1) promotes the growth of breast cancer cells through improving mTOR-mediated mitochondrial biogenesis and function. Biochem Biophys Res Commun.

[41] Mayers JR, Wu C, Clish CB, Kraft P, Torrence ME, Fiske BP (2014). Elevation of circulating branched-chain amino acids is an early event in human pancreatic adenocarcinoma development. Nat Med.

[42] Wang Y, Xiao J, Jiang W, Zuo D, Wang X, Jin Y (2021). BCKDK alters the metabolism of non-small cell lung cancer. Transl Lung Cancer Res.

[43] Luo L, Sun W, Zhu W, Li S, Zhang W, Xu X (2021). BCAT1 decreases the sensitivity of cancer cells to cisplatin by regulating mTOR-mediated autophagy via branched-chain amino acid metabolism. Cell Death Dis.

[44] Huang C, Rajfur Z, Yousefi N, Chen Z, Jacobson K, Ginsberg MH (2009). Talin phosphorylation by Cdk5 regulates Smurf1-mediated talin head ubiquitylation and cell migration. Nat Cell Biol.

[45] Yang L, Jin L, Ke Y, Fan X, Zhang T, Zhang C (2018). E3 ligase Trim21 ubiquitylates and stabilizes keratin 17 to induce STAT3 activation in psoriasis. J Invest Dermatol.

[46] Nguyen JQ, Irby RB (2017). TRIM21 is a novel regulator of Par-4 in colon and pancreatic cancer cells. Cancer Biol Ther.

[47] Wu S, Chen M, Huang J, Zhang F, Lv Z, Jia Y (2021). ORAI2 promotes gastric cancer tumorigenicity and metastasis through PI3K/Akt signaling and MAPK-dependent focal adhesion disassembly. Cancer Res.

[48] Ilić D, Furuta Y, Kanazawa S, Takeda N, Sobue K, Nakatsuji N (1995). Reduced cell motility and enhanced focal adhesion contact formation in cells from FAK-deficient mice. Nature..

[49] Bécam IE, Tanentzapf G, Lepesant JA, Brown NH, Huynh JR (2005). Integrin-independent repression of cadherin transcription by talin during axis formation in *Drosophila*. Nat Cell Biol.

[50] Tang X, Li Q, Li L, Jiang J (2021). Expression of Talin-1 in endometriosis and its possible role in pathogenesis. Reprod Biol Endocrinol.

[51] Tadokoro S, Shattil SJ, Eto K, Tai V, Liddington RC, de Pereda JM (2003). Talin binding to integrin beta tails: a final common step in integrin activation. Science..

[52] Zhong Y, Long T, Gu CS, Tang JY, Gao LF, Zhu JX (2021). MYH9-dependent polarization of ATG9B promotes colorectal cancer metastasis by accelerating focal adhesion assembly. Cell Death Differ.

[53] Jin JK, Tien PC, Cheng CJ, Song JH, Huang C, Lin SH (2015). Talin1 phosphorylation activates beta1 integrins: a novel mechanism to promote prostate cancer bone metastasis. Oncogene..

[54] East MP, Laitinen T, Asquith CRM (2021). BCKDK: an emerging kinase target for metabolic diseases and cancer. Nat Rev Drug Discov.

