# Supplementary figures and images for: BCKDK regulates breast cancer cell adhesion and tumor metastasis by inhibiting TRIM21 ubiquitinate talin1
Source: Cell Death Dis. 2023 Jul 17;14(7):445. doi: 10.1038/s41419-023-05944-4 (PMC10352378; doi:10.1038/s41419-023-05944-4)

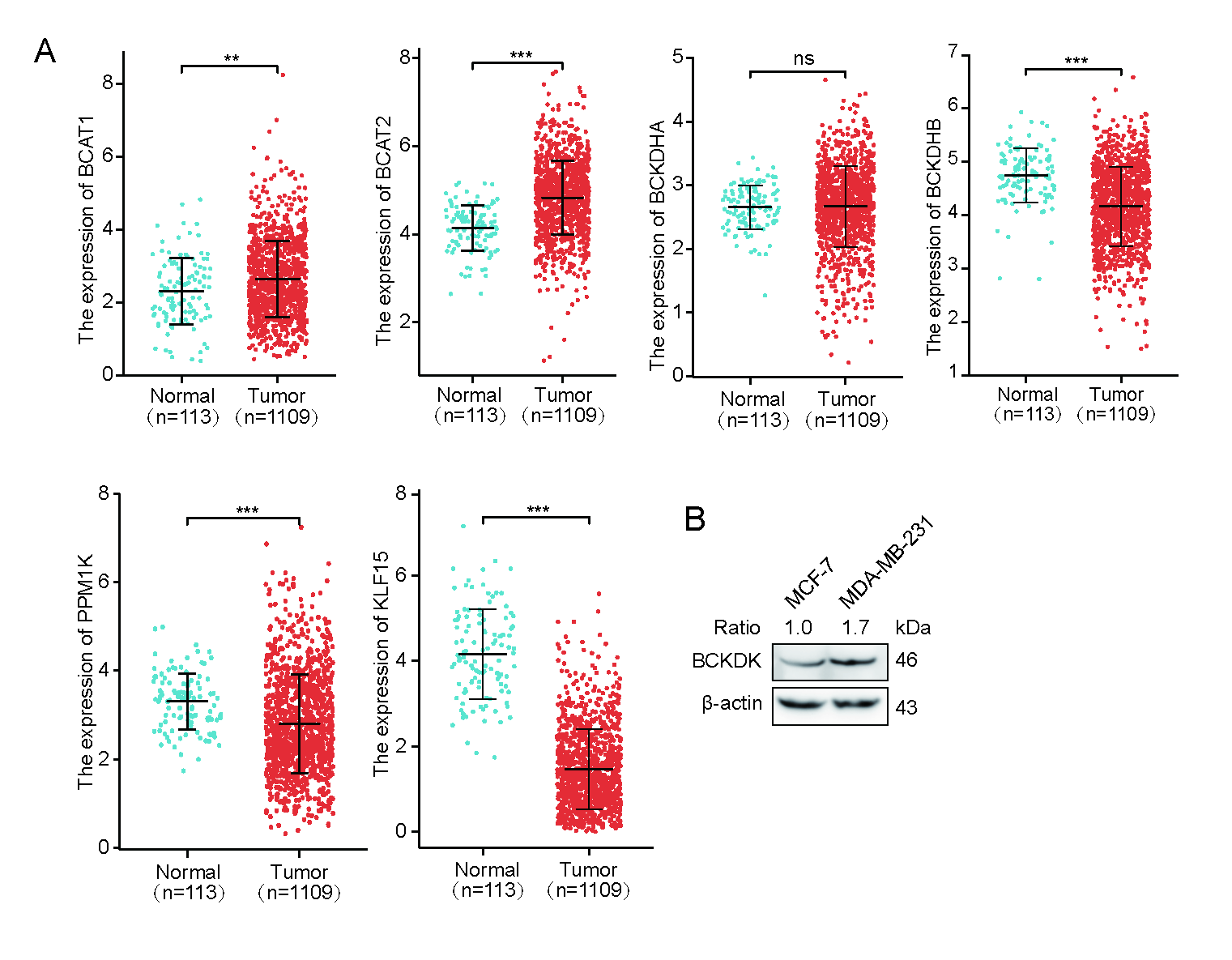

Supplement: Supplementary file 1 — supplementary figure 1 [file 41419_2023_5944_MOESM1_ESM.tif]

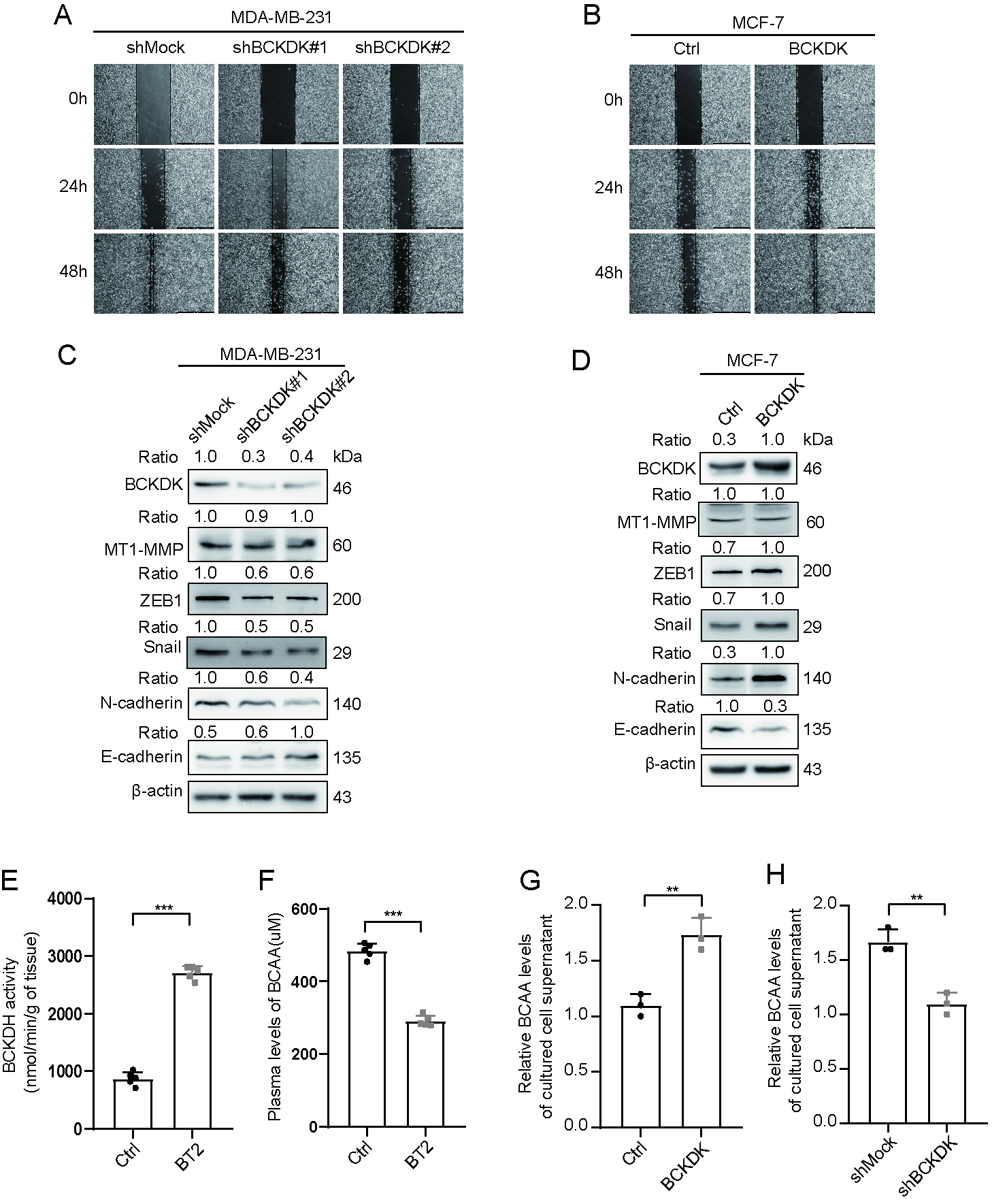

Supplement: Supplementary file 2 — supplementary figure 2 [file 41419_2023_5944_MOESM2_ESM.tif]

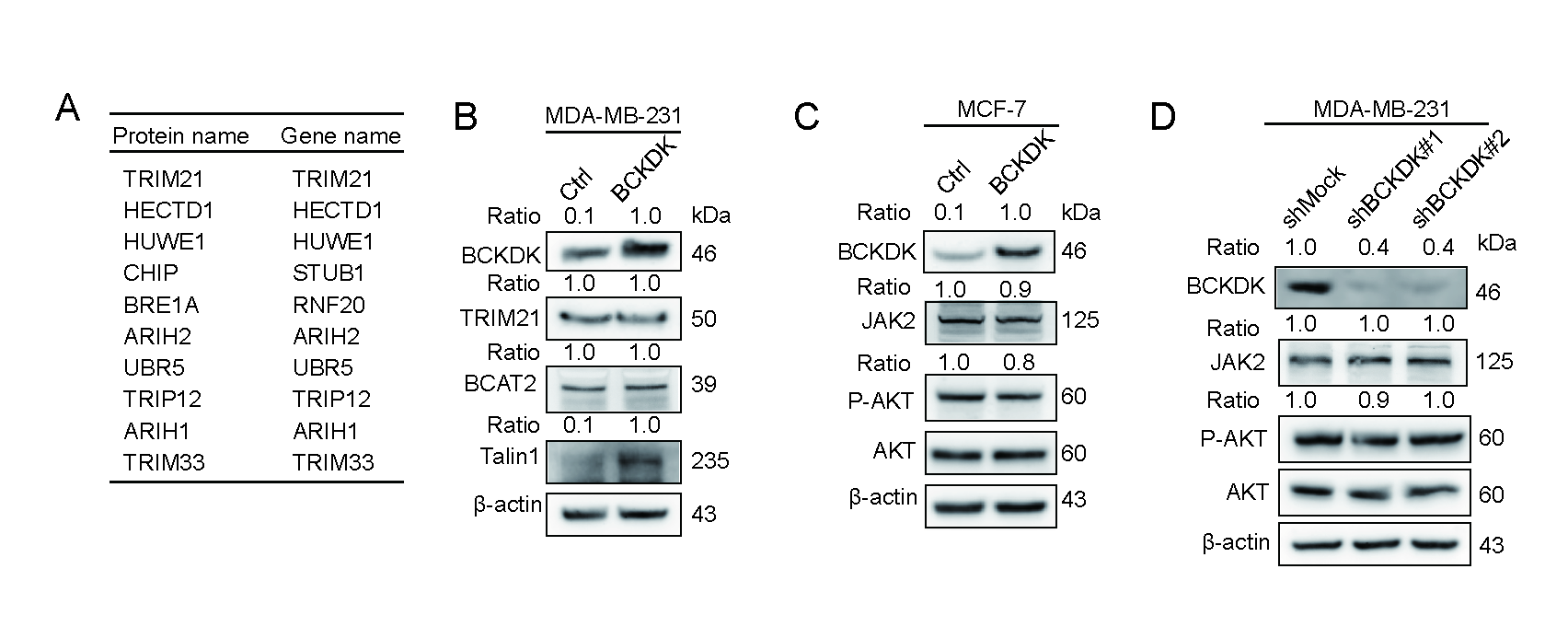

Supplement: Supplementary file 3 — supplementary figure 3 [file 41419_2023_5944_MOESM3_ESM.tif]

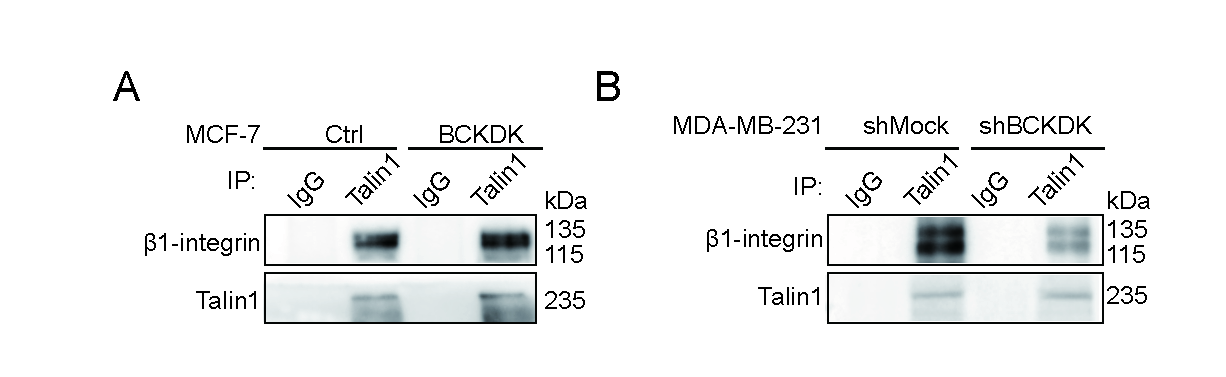

Supplement: Supplementary file 4 — supplementary figure 4 [file 41419_2023_5944_MOESM4_ESM.tif]
