# Supplementary material for: BCKDK regulates breast cancer cell adhesion and tumor metastasis by inhibiting TRIM21 ubiquitinate talin1
Source: Cell Death Dis. 2023 Jul 17;14(7):445. doi: 10.1038/s41419-023-05944-4 (PMC10352378; doi:10.1038/s41419-023-05944-4)
Supplement: Supplementary file 5 — supplementary data file [file 41419_2023_5944_MOESM5_ESM.pdf]

Figure legends of supplementary data

- Fig. S1(A) The mRNA expression of BCAT1, BCAT2, BCKDHA, BCKDHB, PPM1K, KLF15 in normal and tumor tissues in TCGA database, the analysis was performed by R project. Error bars represent the mean ± SD values. (B) The expression of BCKDK in MCF-7 cells and MDA-MB-231 cells was detected by western-blot.
- Fig. S2(A, B) The migration capability was detected by wound healing assays in both cells, Scale bar: 100 μm. (C, D) The expression of EMT-related markers were detected by western-blot. (E, F) Effects of BT2 on circulating branched-chain amino acids (BCAA) and liver BCKDH activity. (G, H) The concentration of BCAA in the cellular supernatant.
- Fig. S3 (A) A list of potential E3 ubiquitin ligases that interacted with talin1 by performing Co-IP and mass spectrometry assays. (B) The expression of TRIM21 in MDA-MB-231 cells was detected by western-blot. (C, D) The expression of JAK2, P-AKT, AKT were detected by western-blot.
- Fig. S4(A, B) The immunoprecipitation of talin1 and β1-integrin was detected in these cell lines.

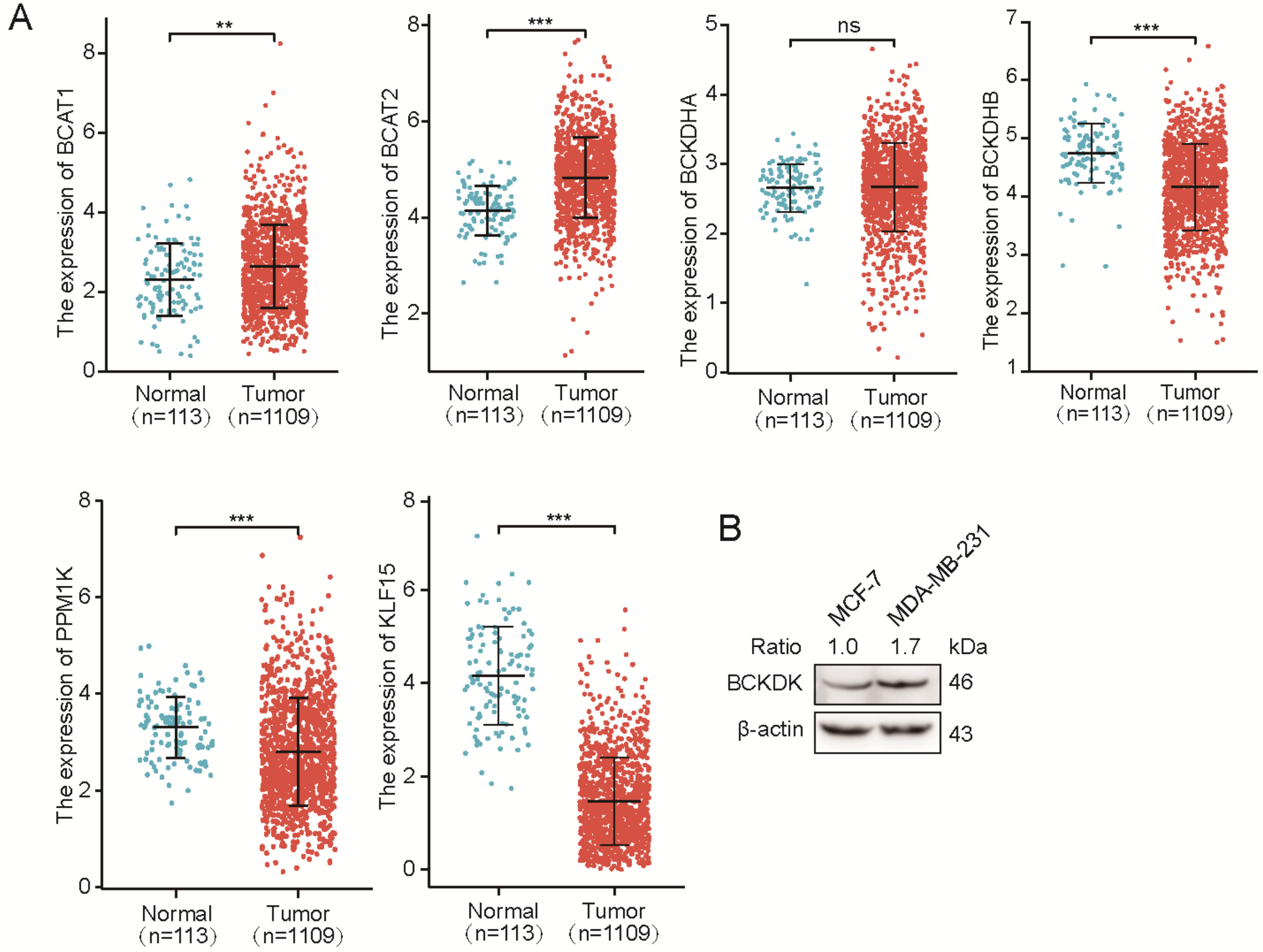

Fig. S1(A) The mRNA expression of BCAT1, BCAT2, BCKDHA, BCKDHB, PPM1K, KLF15 in normal and tumor tissues in TCGA database, the analysis was performed by R project. Error bars represent the mean ± SD values. (B) The expression of BCKDK in MCF-7 cells and MDA-MB-231 cells was detected by western-blot.

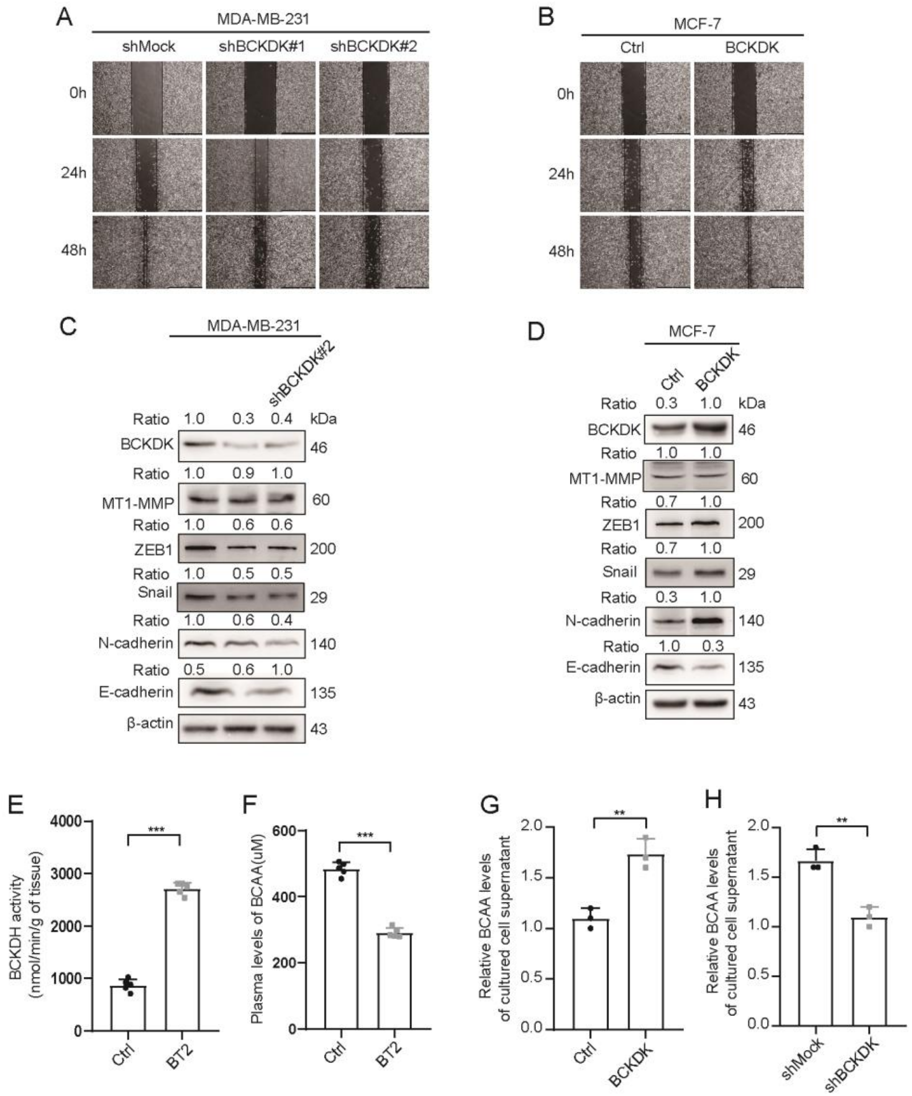

Fig. S2(A, B) The migration capability was detected by wound healing assays in both cells, Scale bar: 100 μm. (C, D) The expression of EMT-related markers were detected by western-blot. (E, F) Effects of BT2 on circulating branched-chain amino acids (BCAA) and liver BCKDH activity. (G, H) The concentration of BCAA in the cellular supernatant.

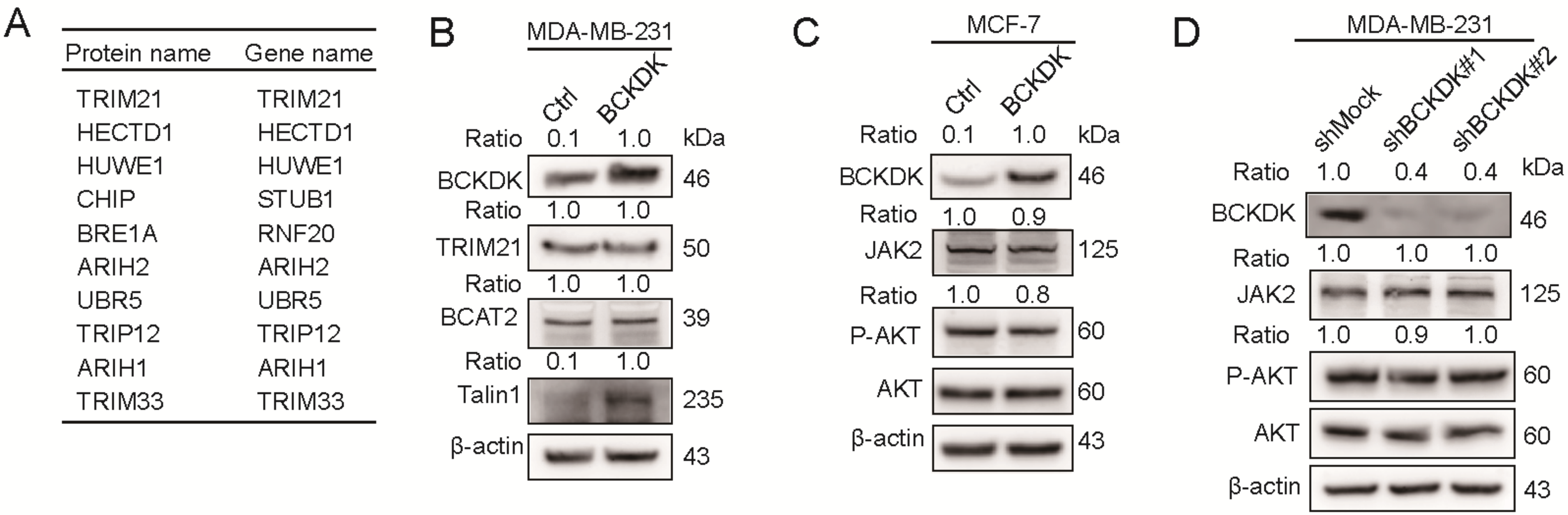

Fig. S3 (A) A list of potential E3 ubiquitin ligases that interacted with talin1 by performing Co-IP and mass spectrometry assays. (B) The expression of TRIM21 in MDA-MB-231 cells was detected by western-blot. (C, D) The expression of JAK2, P-AKT, AKT were detected by western-blot.

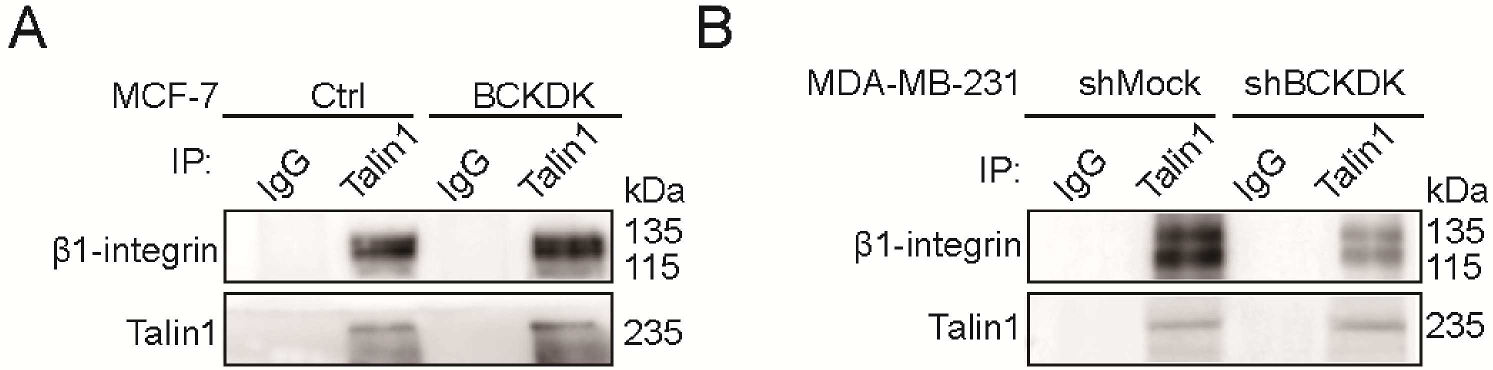

Fig. S4(A, B) The immunoprecipitation of talin1 and β1-integrin was detected in these cell lines.
