# Supplementary material for: BCKDK regulates breast cancer cell adhesion and tumor metastasis by inhibiting TRIM21 ubiquitinate talin1
Source: Cell Death Dis. 2023 Jul 17;14(7):445. doi: 10.1038/s41419-023-05944-4 (PMC10352378; doi:10.1038/s41419-023-05944-4)
Supplement: Supplementary file 6 — Original Data File [file 41419_2023_5944_MOESM6_ESM.pdf]

FIG.1E

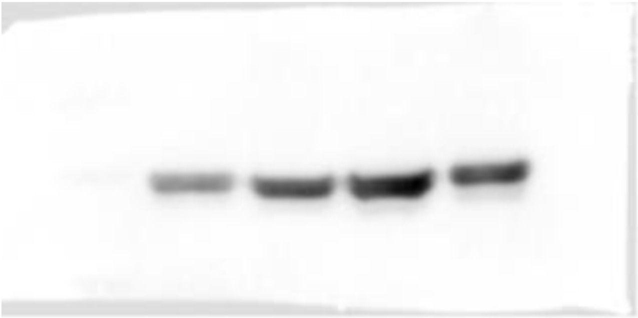

BCKDK

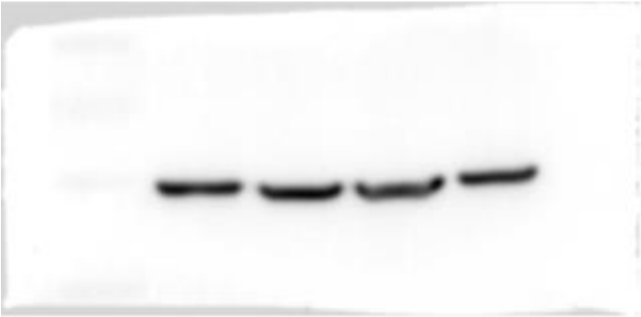

β-actin

FIG.S1B

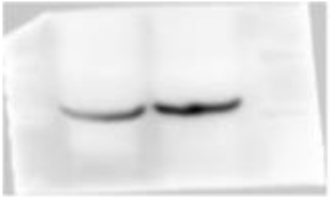

BCKDK

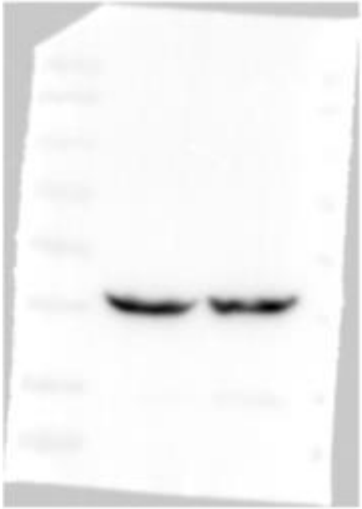

β-actin

FIG.2A

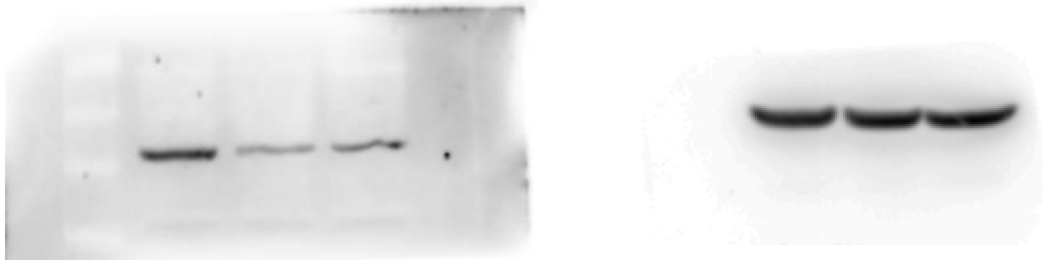

BCKDK β-actin

FIG.2B

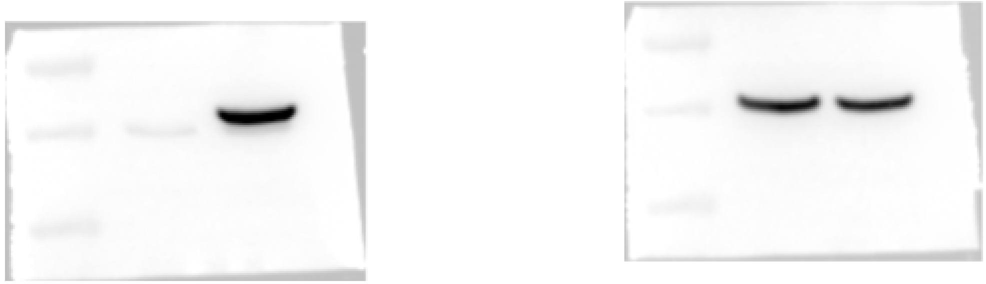

BCKDK β-actin

FIG.2I

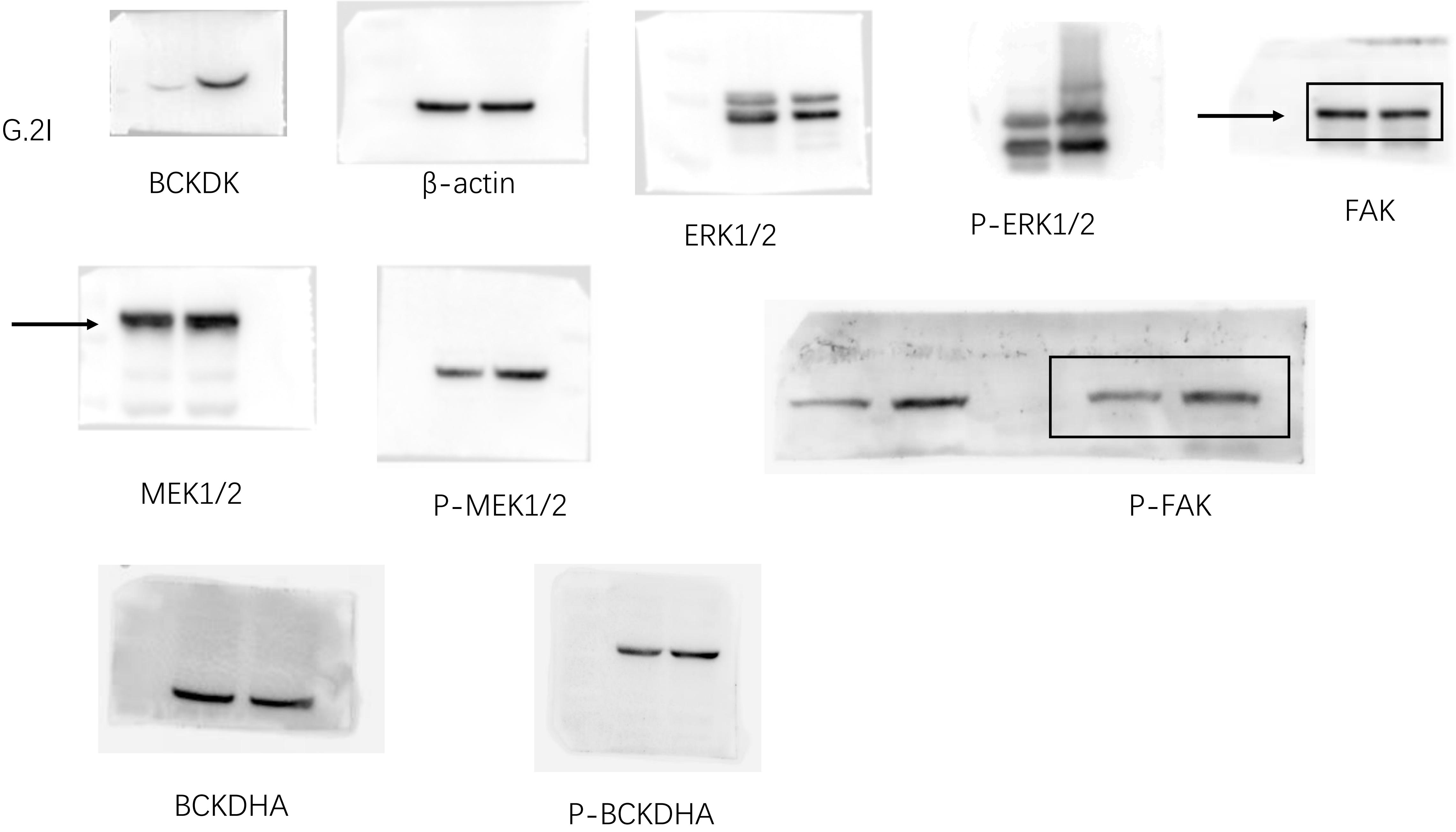
BCKDK
β-actin
ERK1/2
P-ERK1/2
FAK
MEK1/2
P-MEK1/2
P-FAK
BCKDHA
P-BCKDHA

FIG.2J

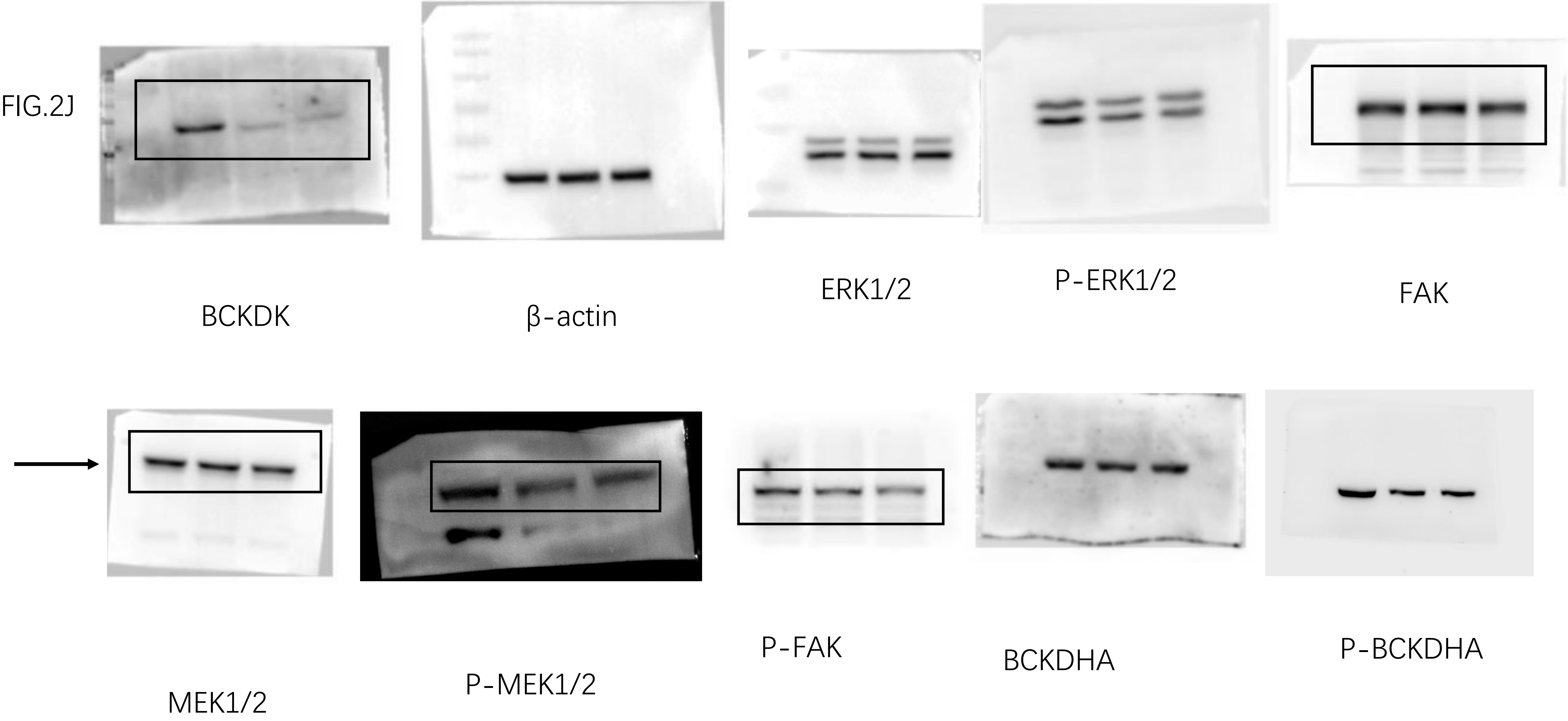
BCKDK
β-actin
ERK1/2
P-ERK1/2
FAK
MEK1/2
P-MEK1/2
P-FAK
BCKDHA
P-BCKDHA

FIG.S2C

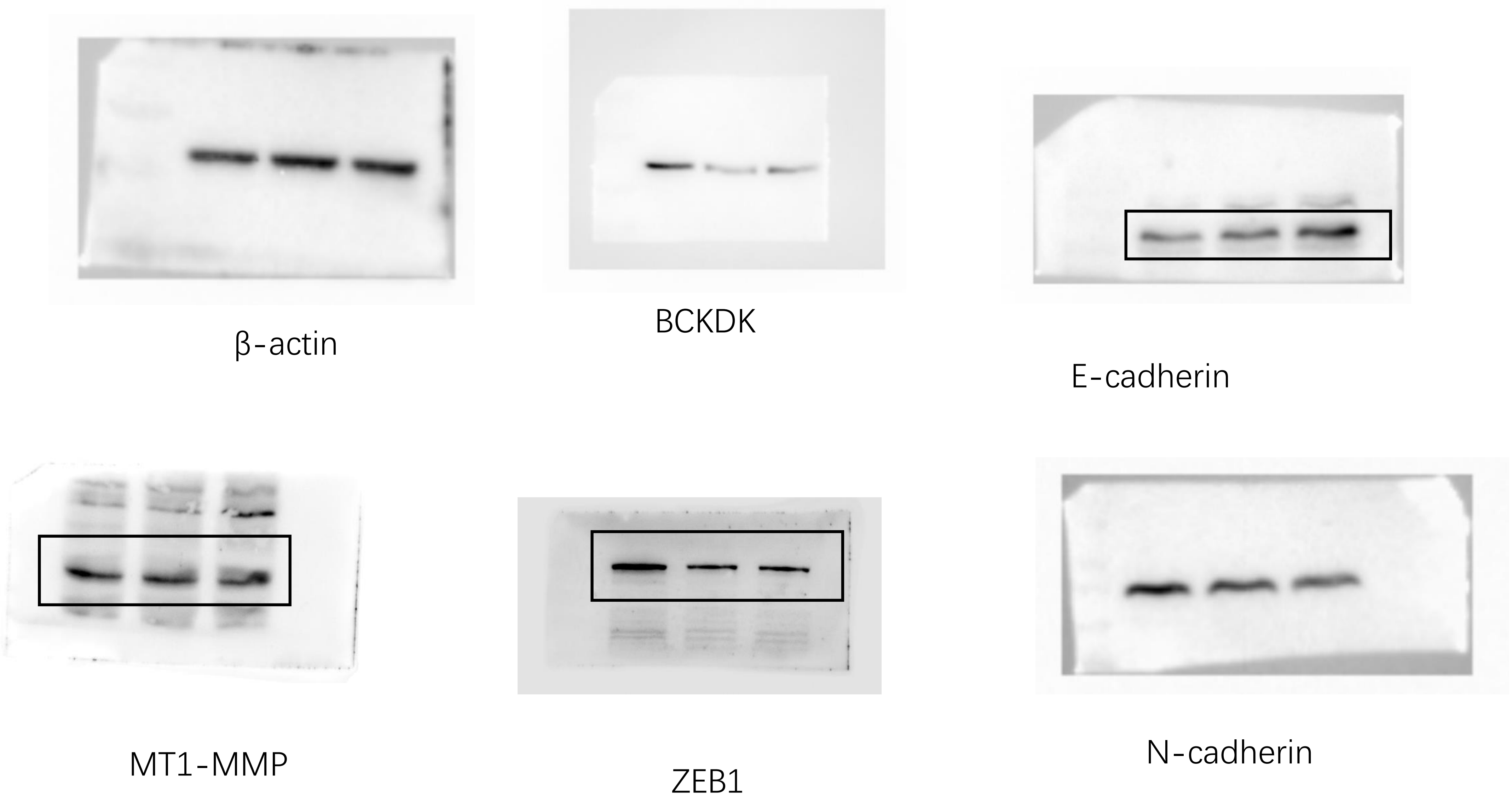

FIG.S2D

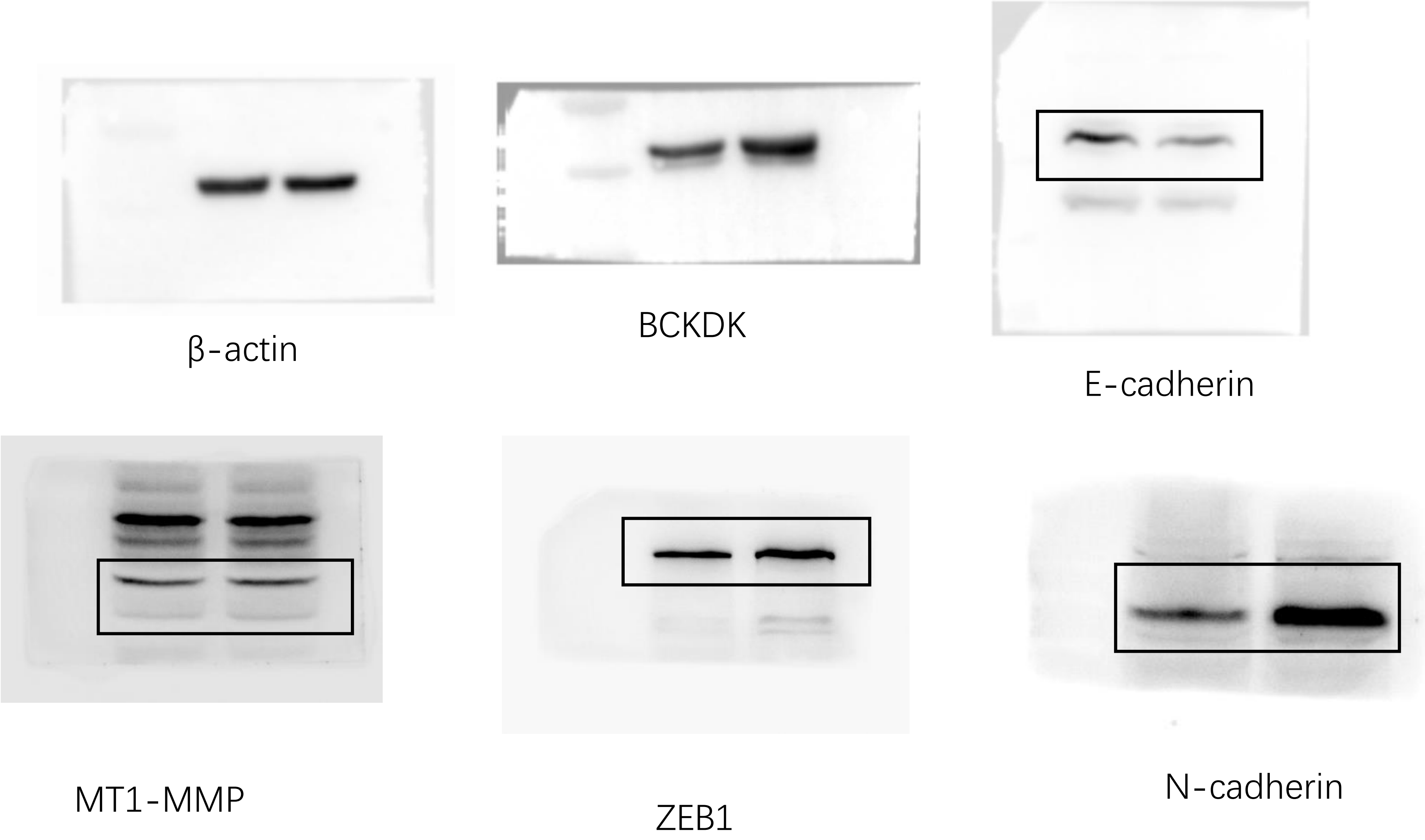

FIG.3A

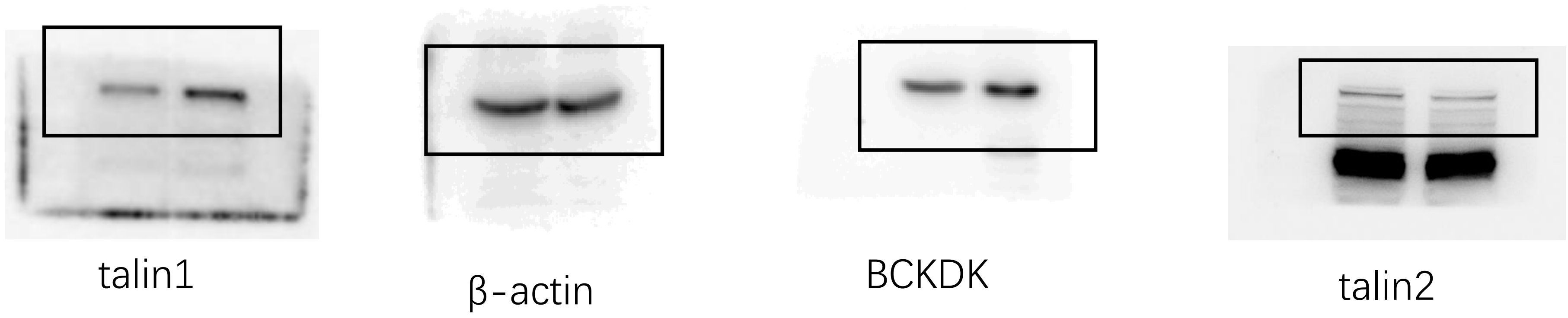

FIG.3B

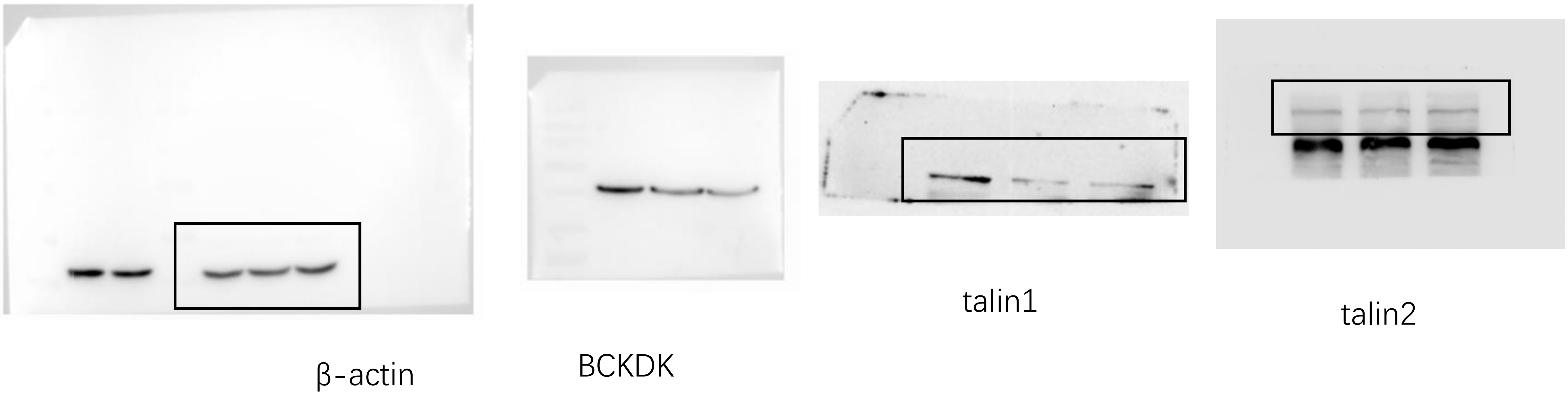

FIG.3D

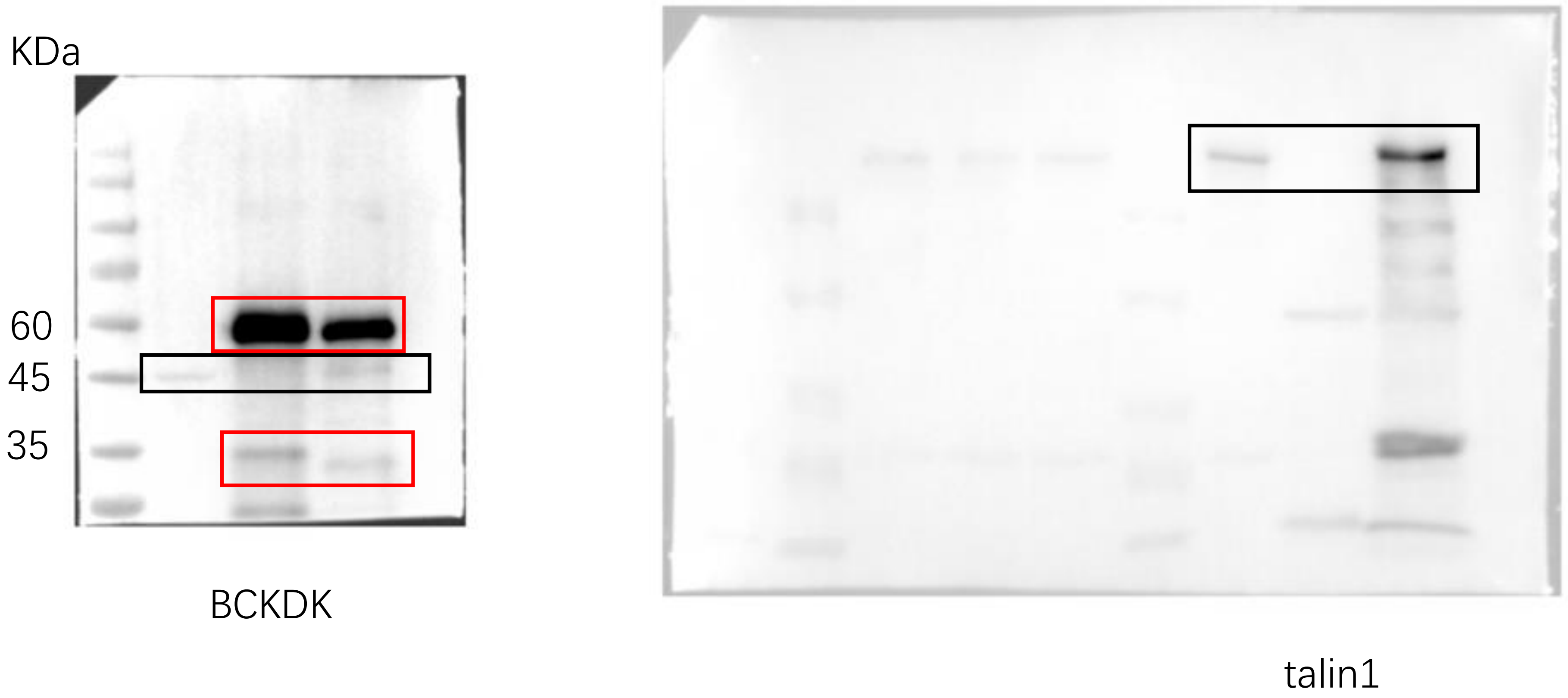

FIG.3E

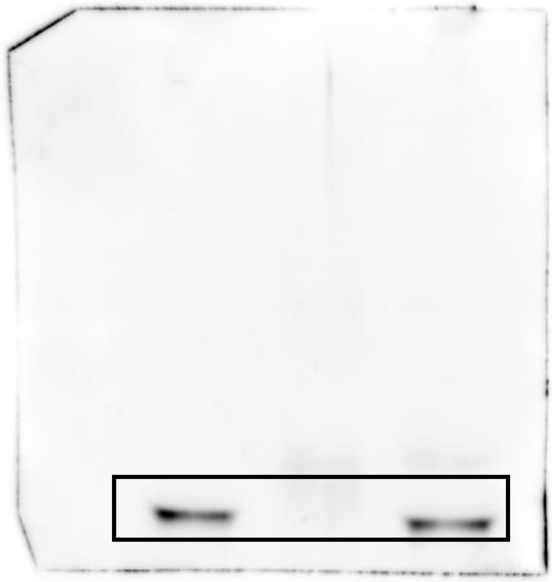

BCKDK

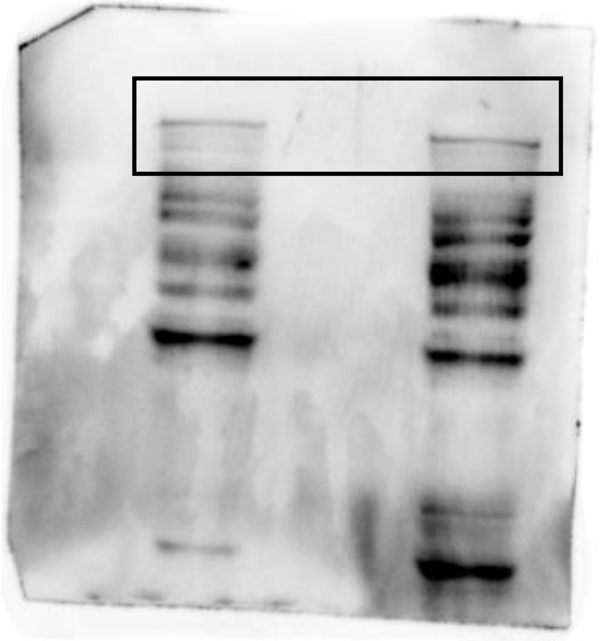

talin1

FIG.3F

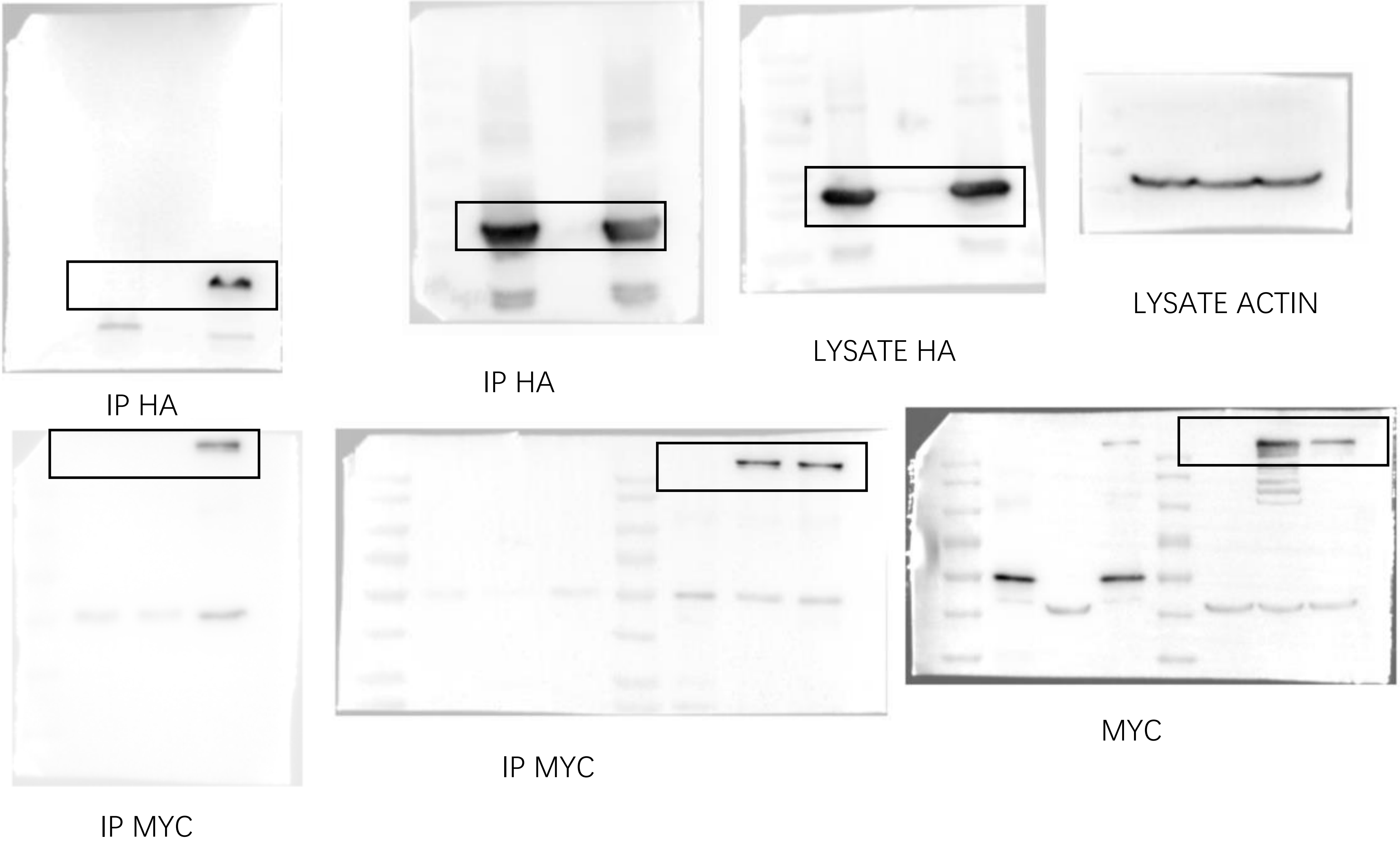

FIG.3G

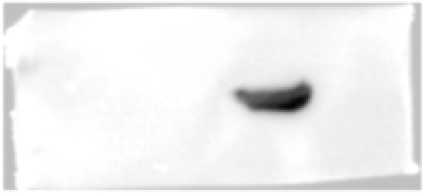

FLAG

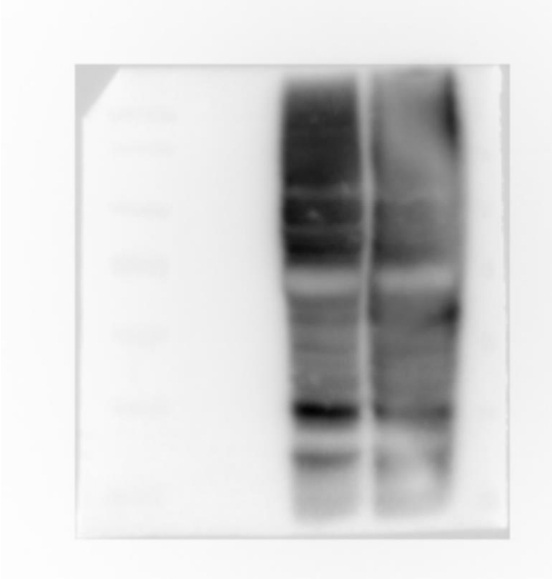

HA

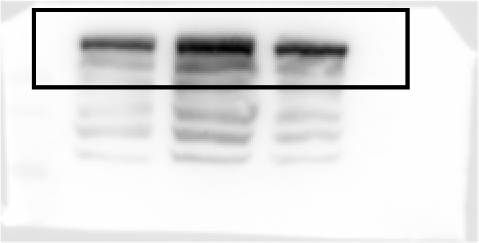

LYSATE MYC

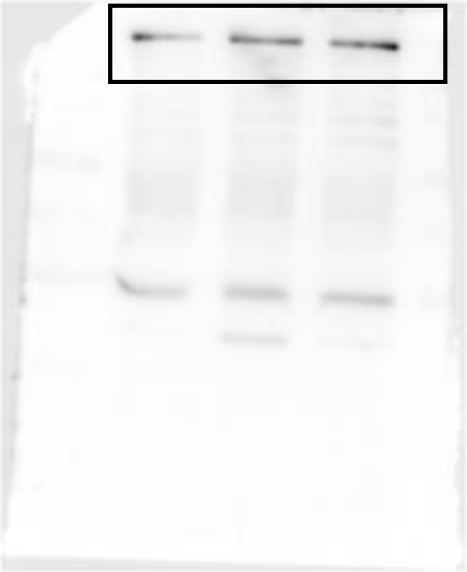

IP MYC

FIG.4A

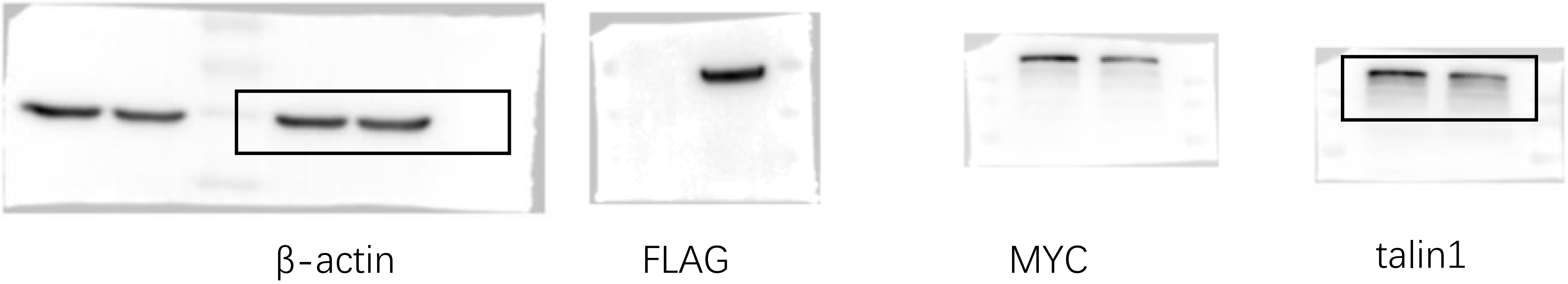

FIG.4B

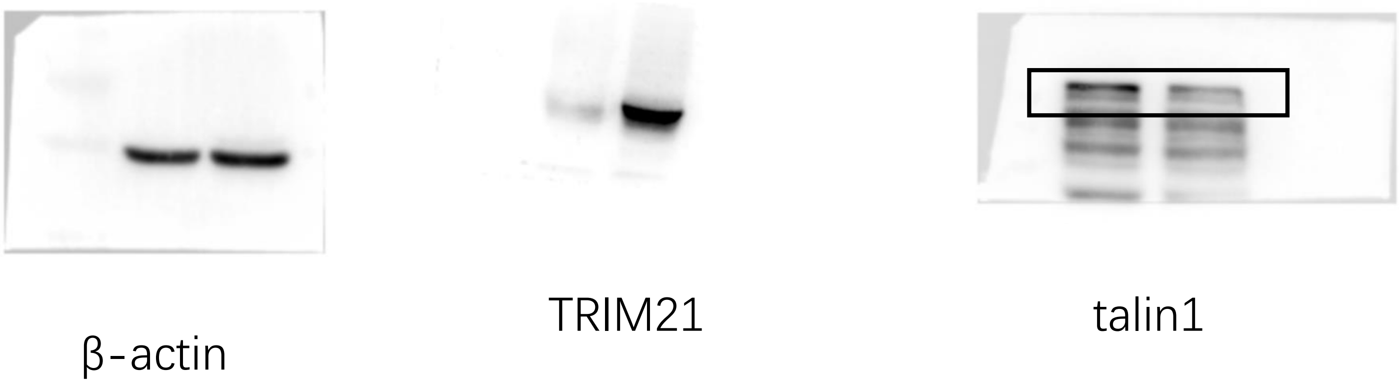

FIG.4C

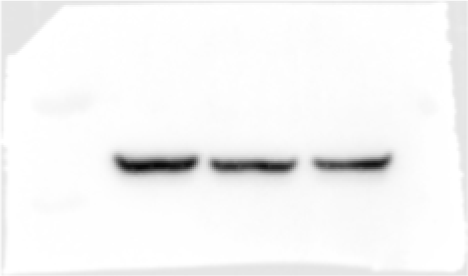

β-actin

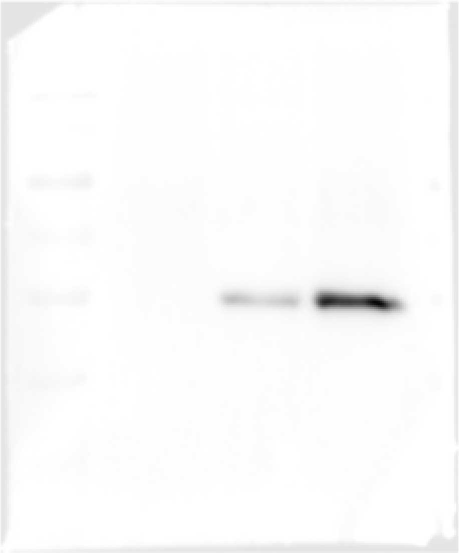

IP FLAG

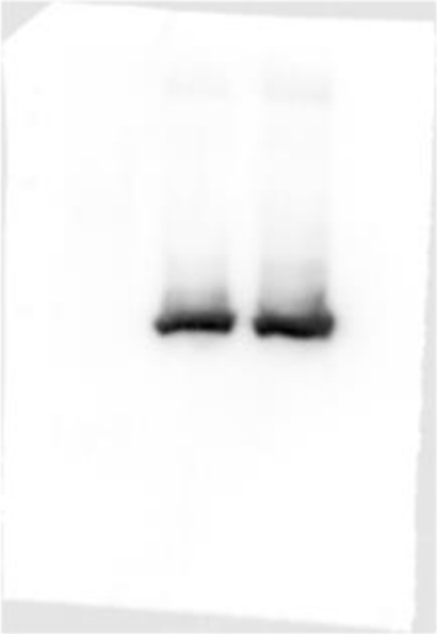

LYSATE FLAG

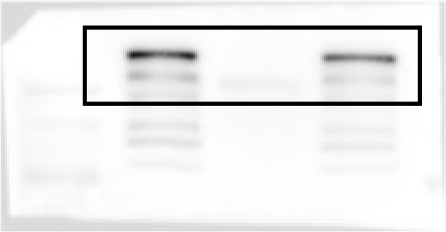

LYSATE MYC

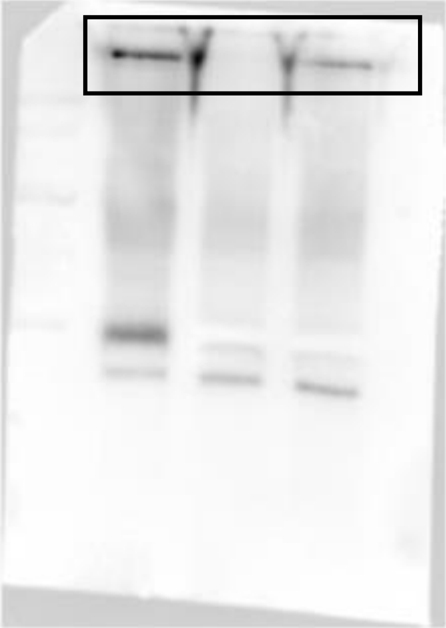

IP MYC

FIG.4D

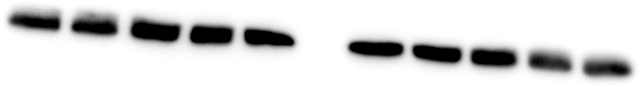

β-actin

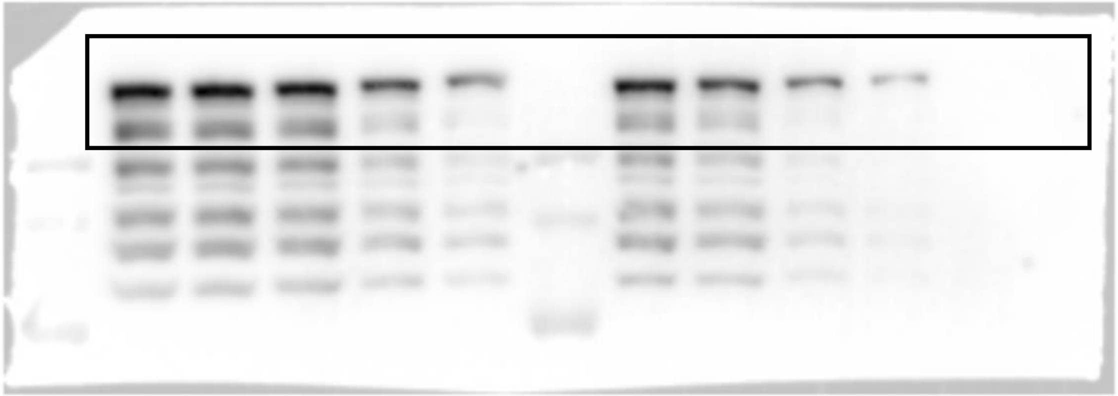

talin1

FIG.4E

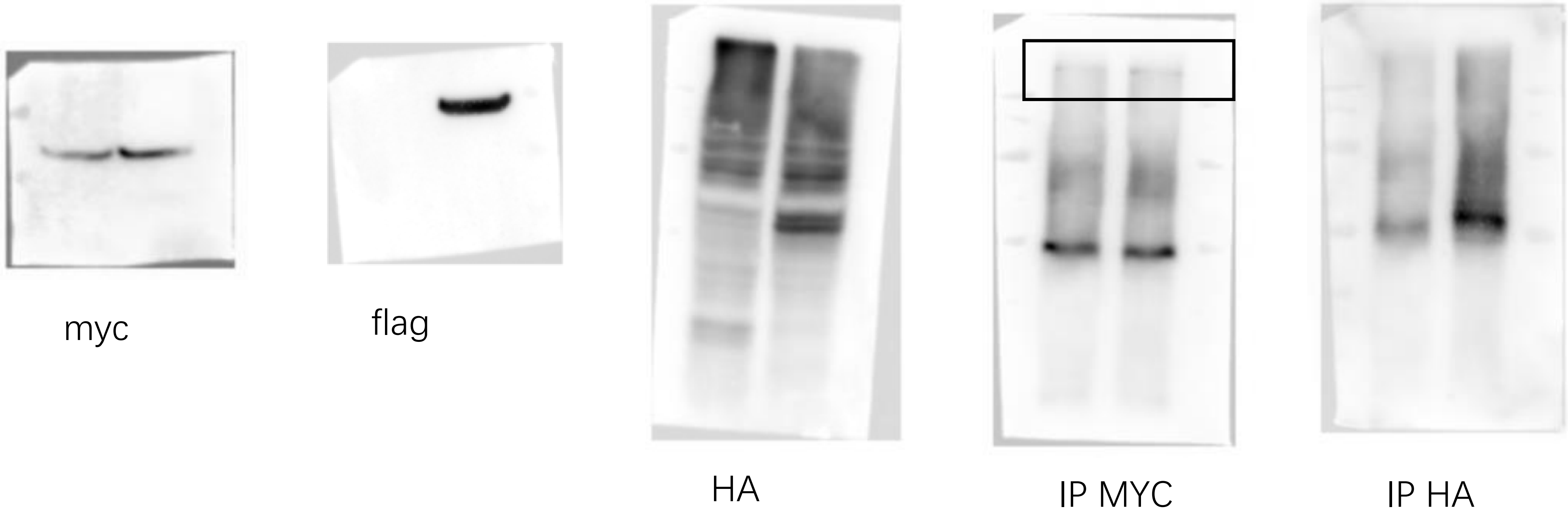

FIG.4F

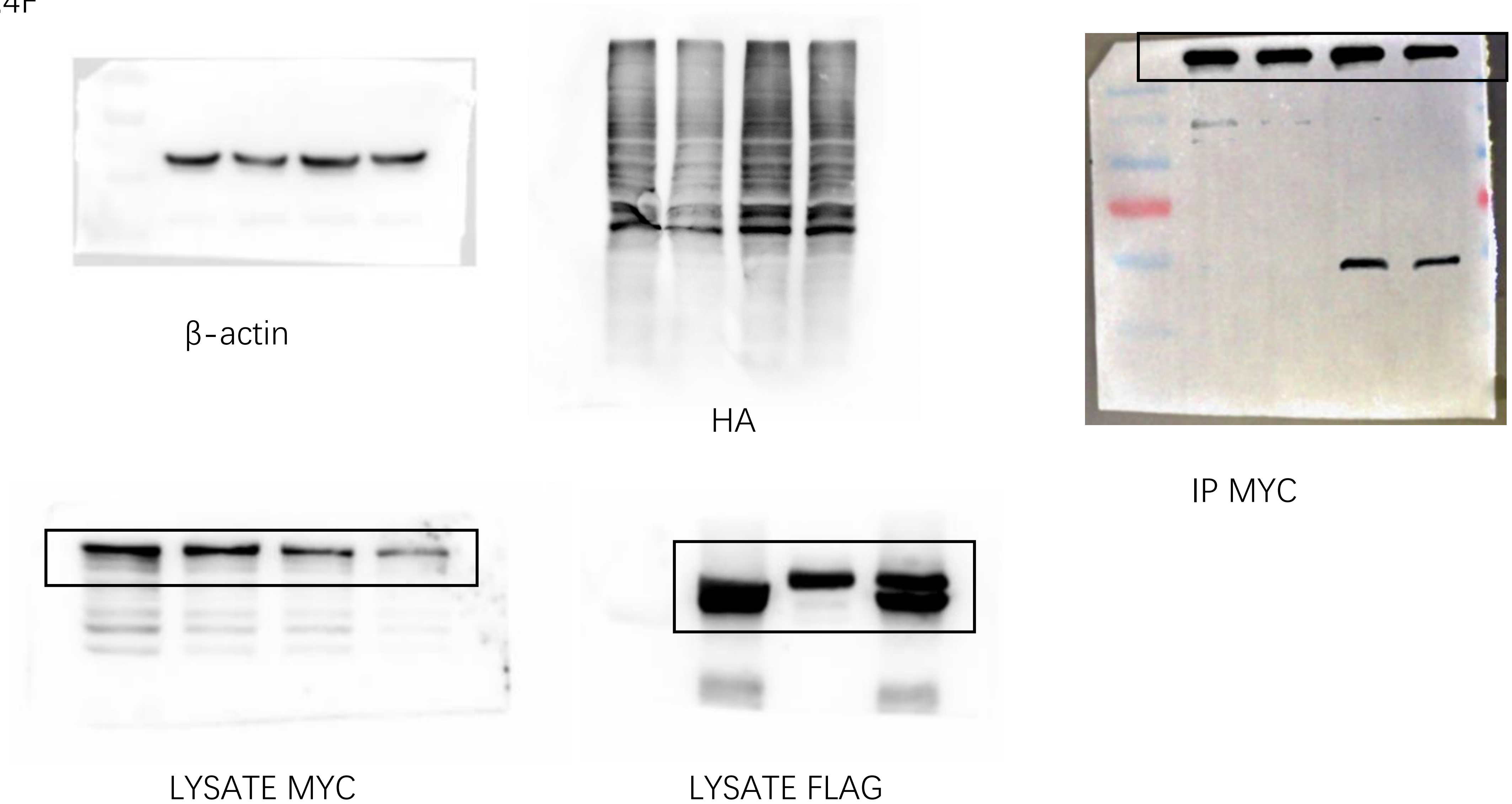

FIG.4G

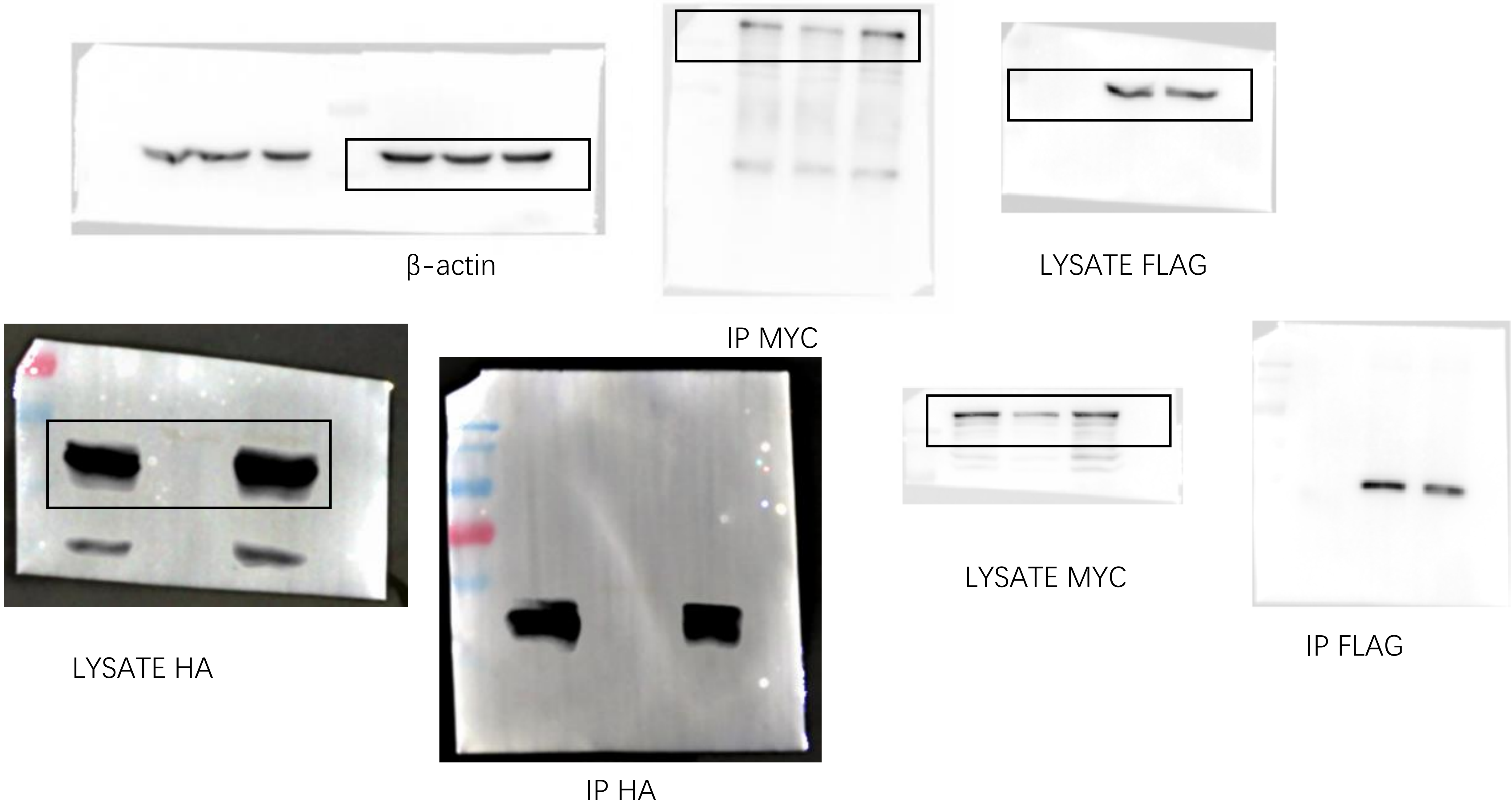

FIG.S3B

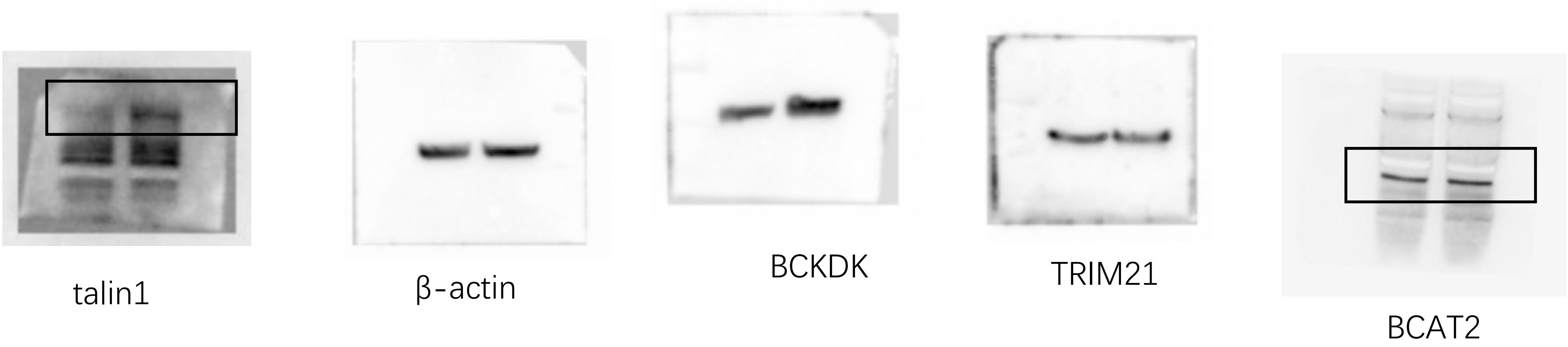

FIG.S3C

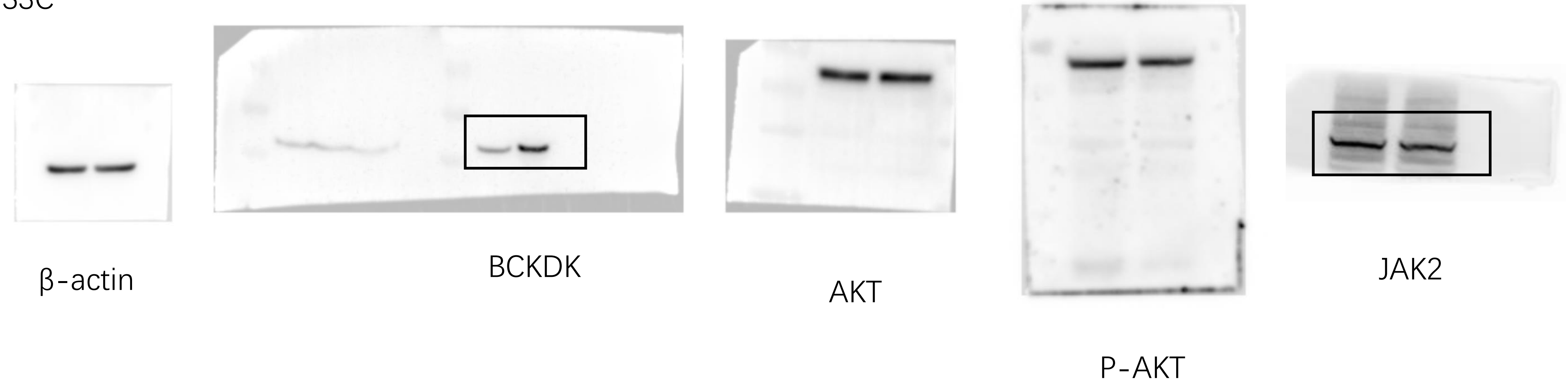

FIG.S3D

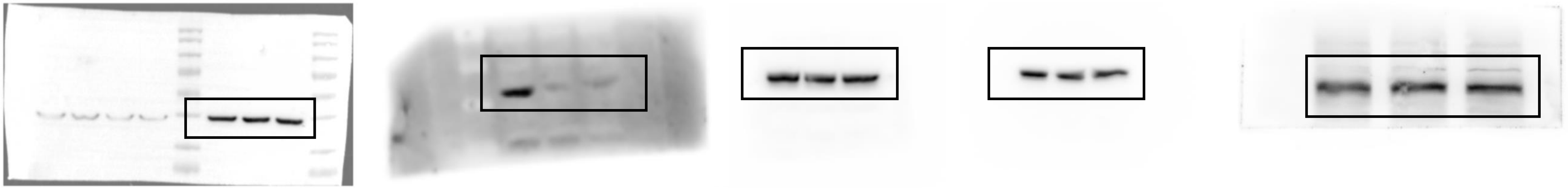

JAK2

β-actin

AKT

P-AKT

BCKDK

FIG.5A

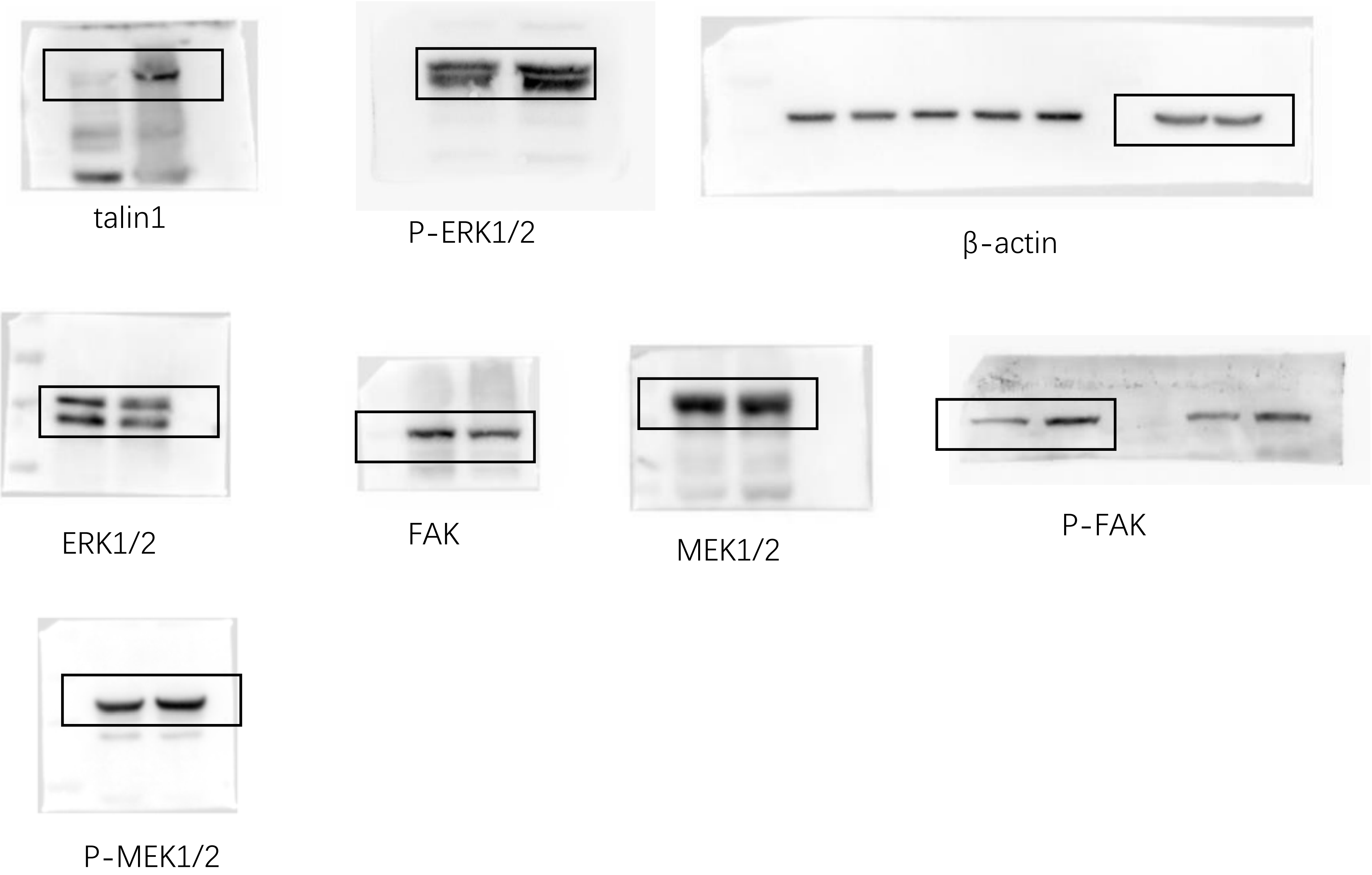

FIG.5B

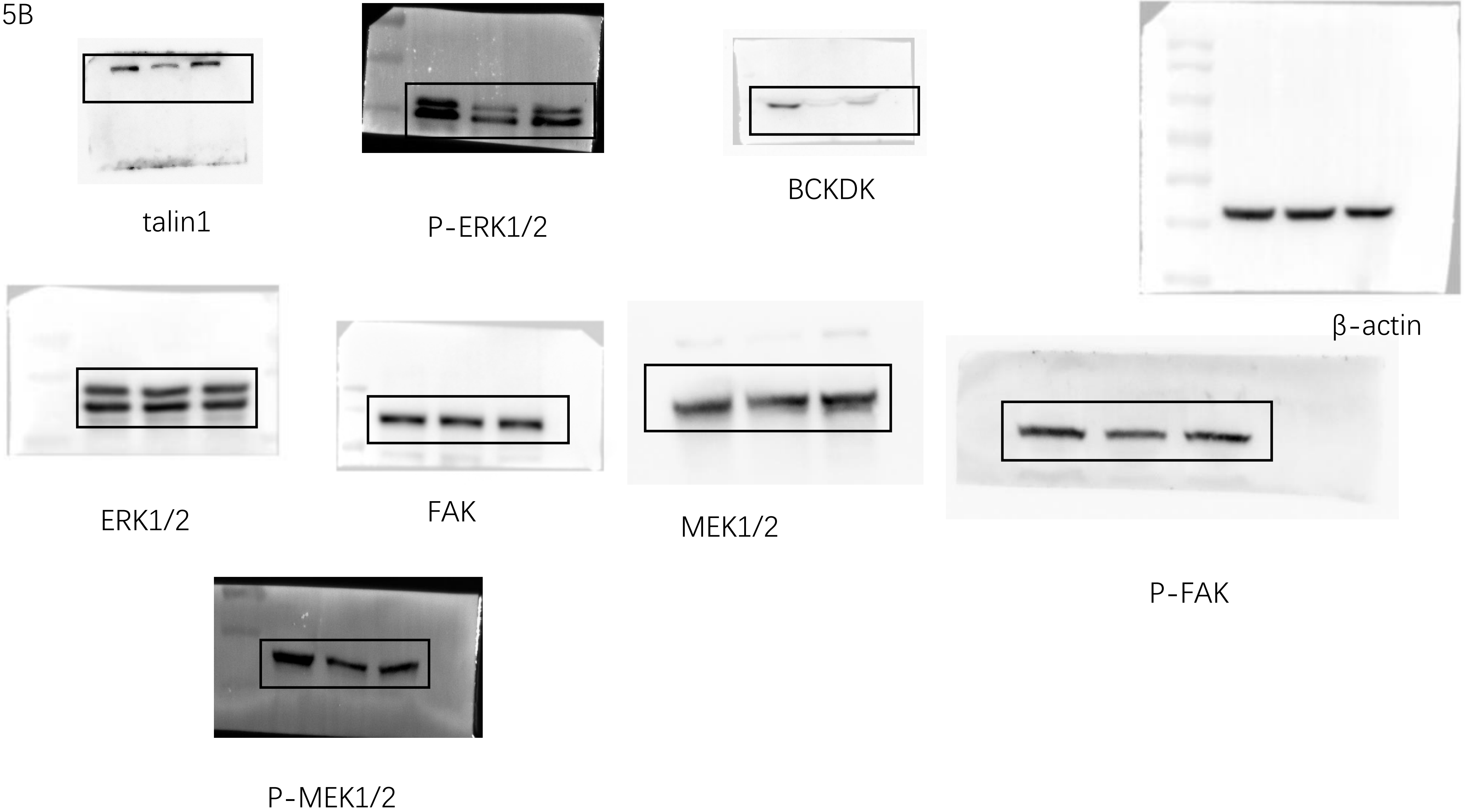

FIG.S4A

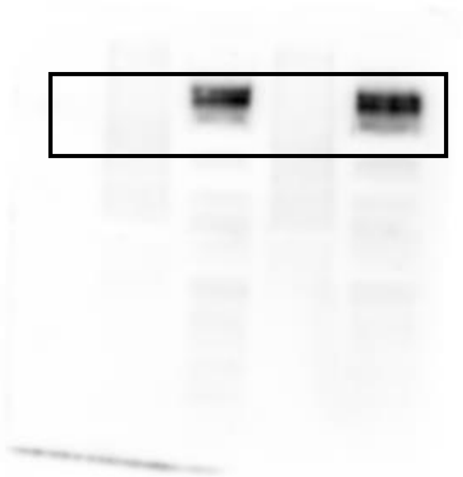

β1-integrin

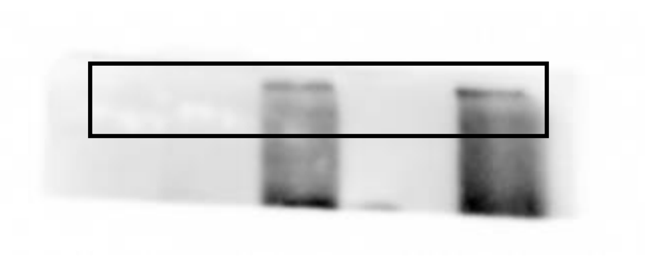

talin1

FIG.S4B

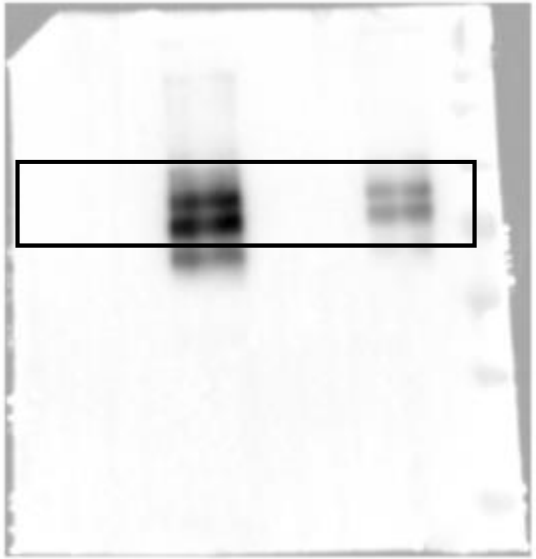

β1-integrin

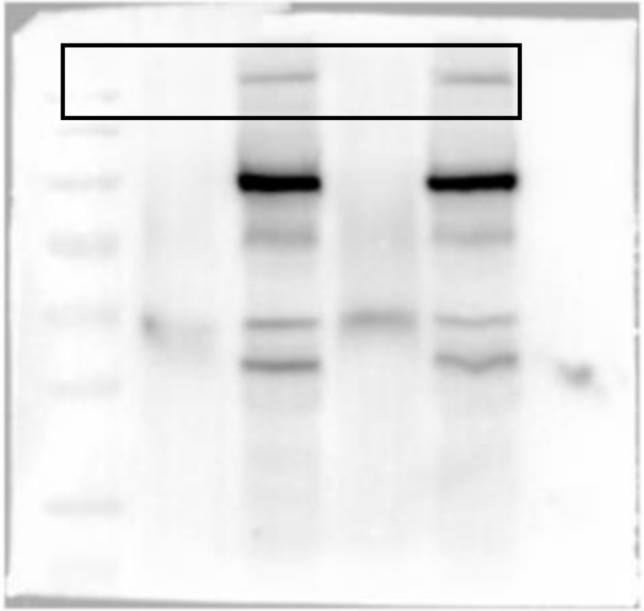

talin1
